# Supplementary material for: A Comprehensive Evolutionary Study of Chloroplast RNA Editing in Gymnosperms: A Novel Type of G-to-A RNA Editing Is Common in Gymnosperms
Source: Int J Mol Sci. 2022 Sep 16;23(18):10844. doi: 10.3390/ijms231810844 (PMC9505161; doi:10.3390/ijms231810844)
Supplement: Supplementary file 1 [file ijms-23-10844-s001.zip › fig S1.pdf]

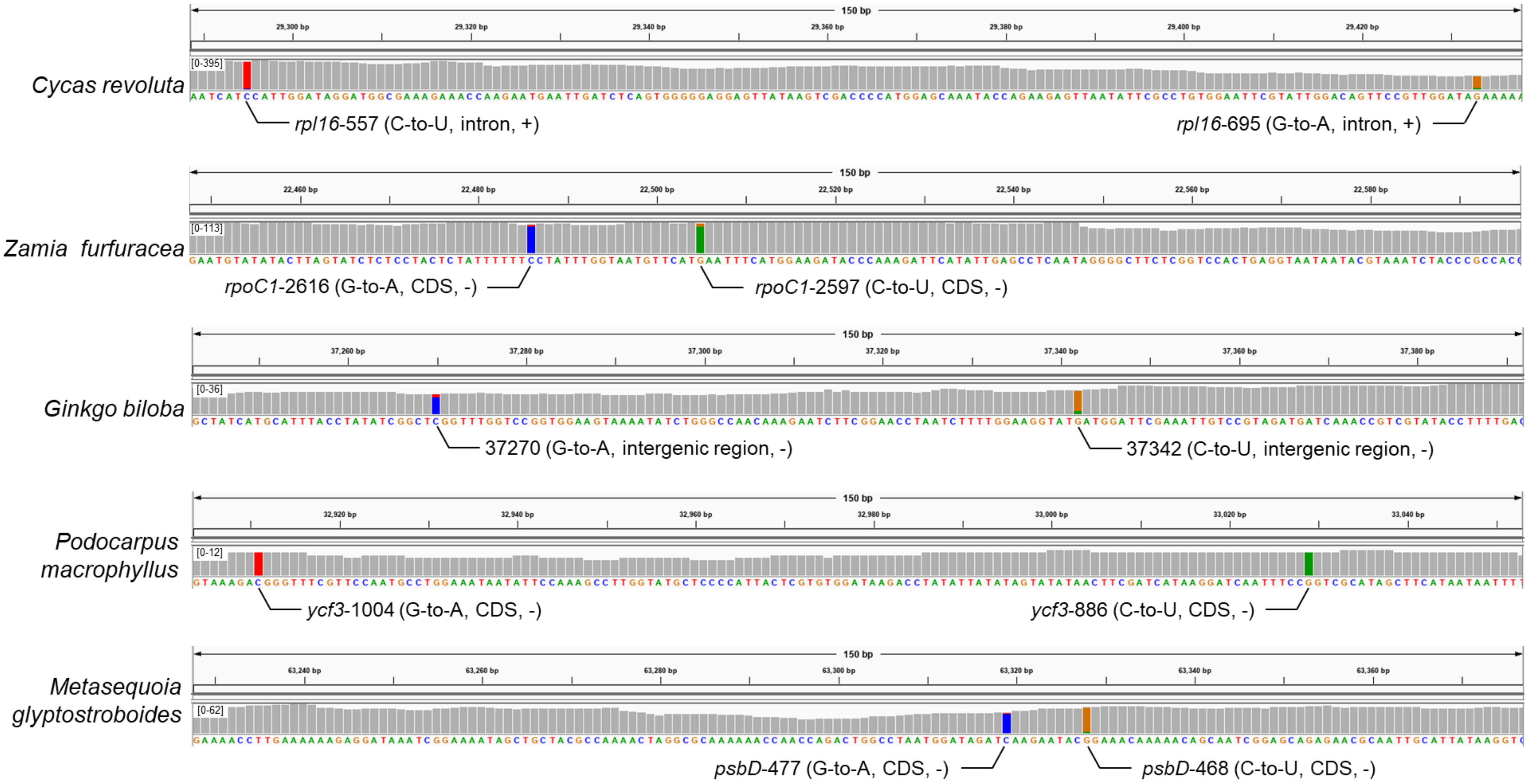

Figure S1. IGV (Integrative Genomics Viewer) views of both types of RNA editing sites occurred in 150-bp region. The colored bars indicate the nucleotide composition of the editing positions from the mapping RNA-seq reads, and numbers on the left-top show read coverage. The genic or plastomic positions of RNA editing sites were shown below the editing sites, including the editing types, the plastomic regions and the strand in the parentheses.
