# Supplementary material for: A Comprehensive Evolutionary Study of Chloroplast RNA Editing in Gymnosperms: A Novel Type of G-to-A RNA Editing Is Common in Gymnosperms
Source: Int J Mol Sci. 2022 Sep 16;23(18):10844. doi: 10.3390/ijms231810844 (PMC9505161; doi:10.3390/ijms231810844)
Supplement: Supplementary file 1 [file ijms-23-10844-s001.zip › fig S2.pdf]

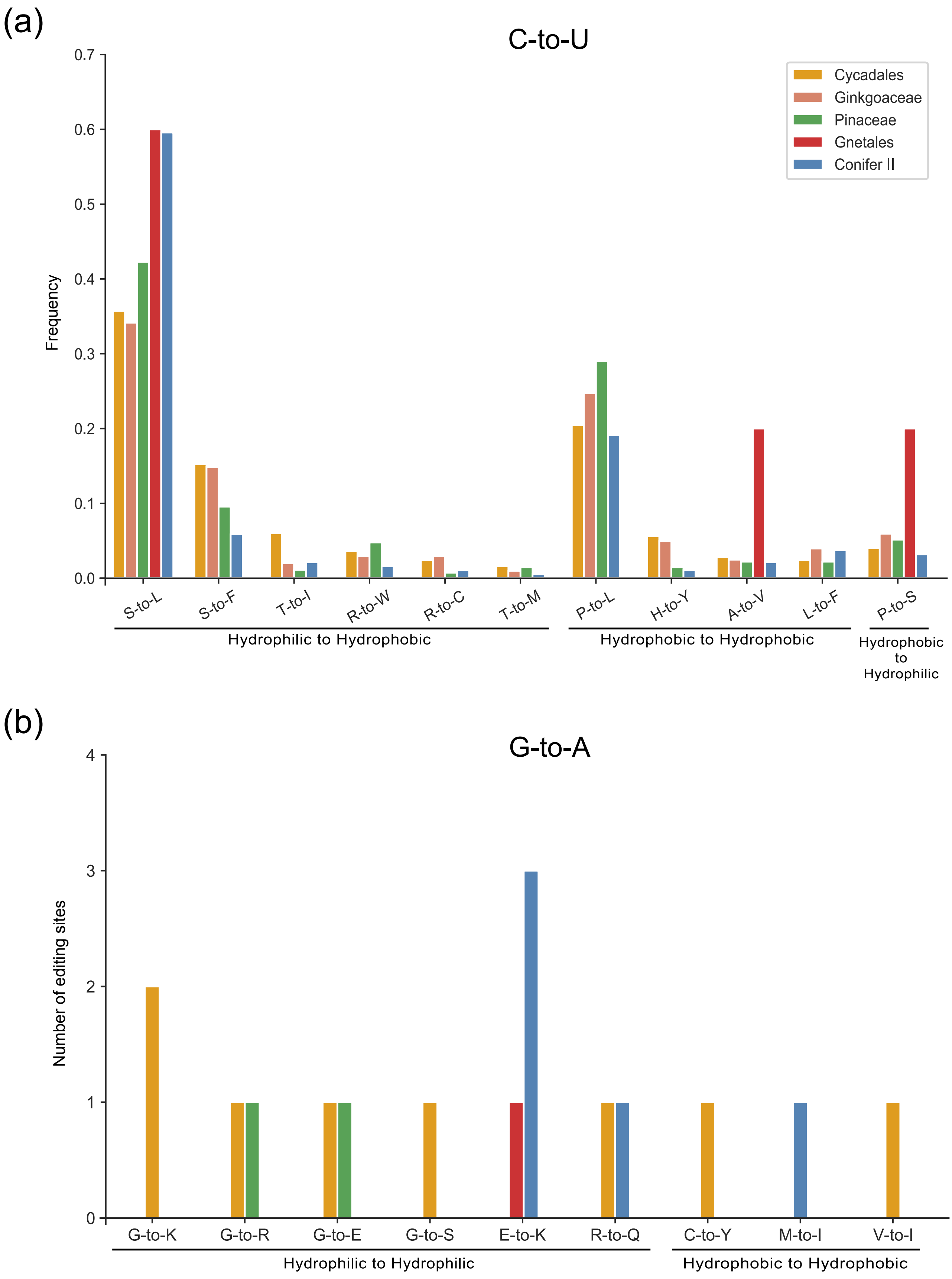

Figure S2. The hydrophilicity or hydrophobicity changes associated with amino acid changes that occurred in nonsilenced editing in different gymnosperm lineages. (a) C-to-U; (b) G-to-A.
