# Supplementary material for: A Comprehensive Evolutionary Study of Chloroplast RNA Editing in Gymnosperms: A Novel Type of G-to-A RNA Editing Is Common in Gymnosperms
Source: Int J Mol Sci. 2022 Sep 16;23(18):10844. doi: 10.3390/ijms231810844 (PMC9505161; doi:10.3390/ijms231810844)
Supplement: Supplementary file 1 [file ijms-23-10844-s001.zip › fig S3.pdf]

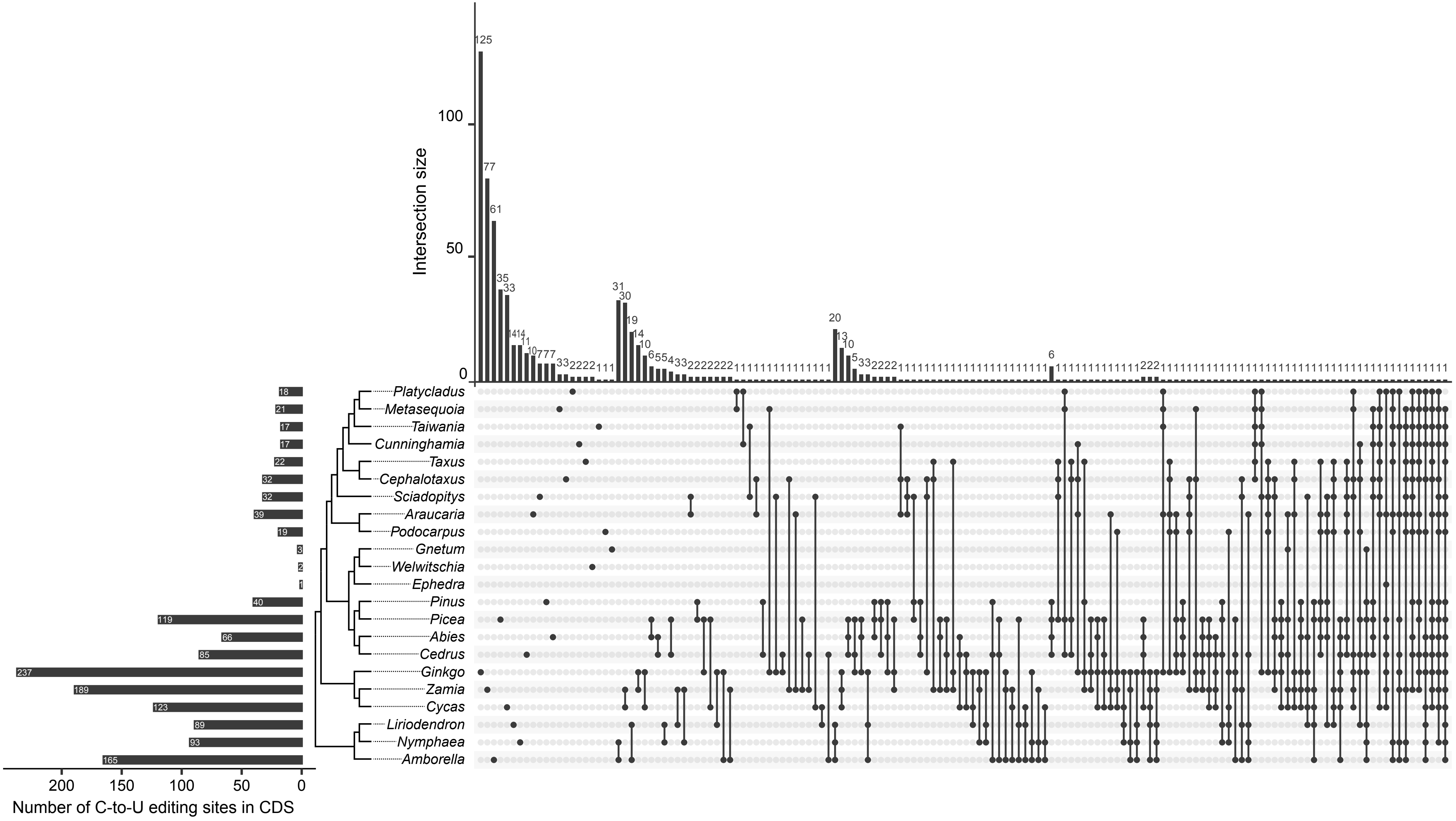

Figure S3. UpsetR plot illustrating the intersections of chloroplast C-to-U RNA editing sites in 19 sampled gymnosperms.
