# Supplementary material for: A Comprehensive Evolutionary Study of Chloroplast RNA Editing in Gymnosperms: A Novel Type of G-to-A RNA Editing Is Common in Gymnosperms
Source: Int J Mol Sci. 2022 Sep 16;23(18):10844. doi: 10.3390/ijms231810844 (PMC9505161; doi:10.3390/ijms231810844)
Supplement: Supplementary file 1 [file ijms-23-10844-s001.zip › fig S4.pdf]

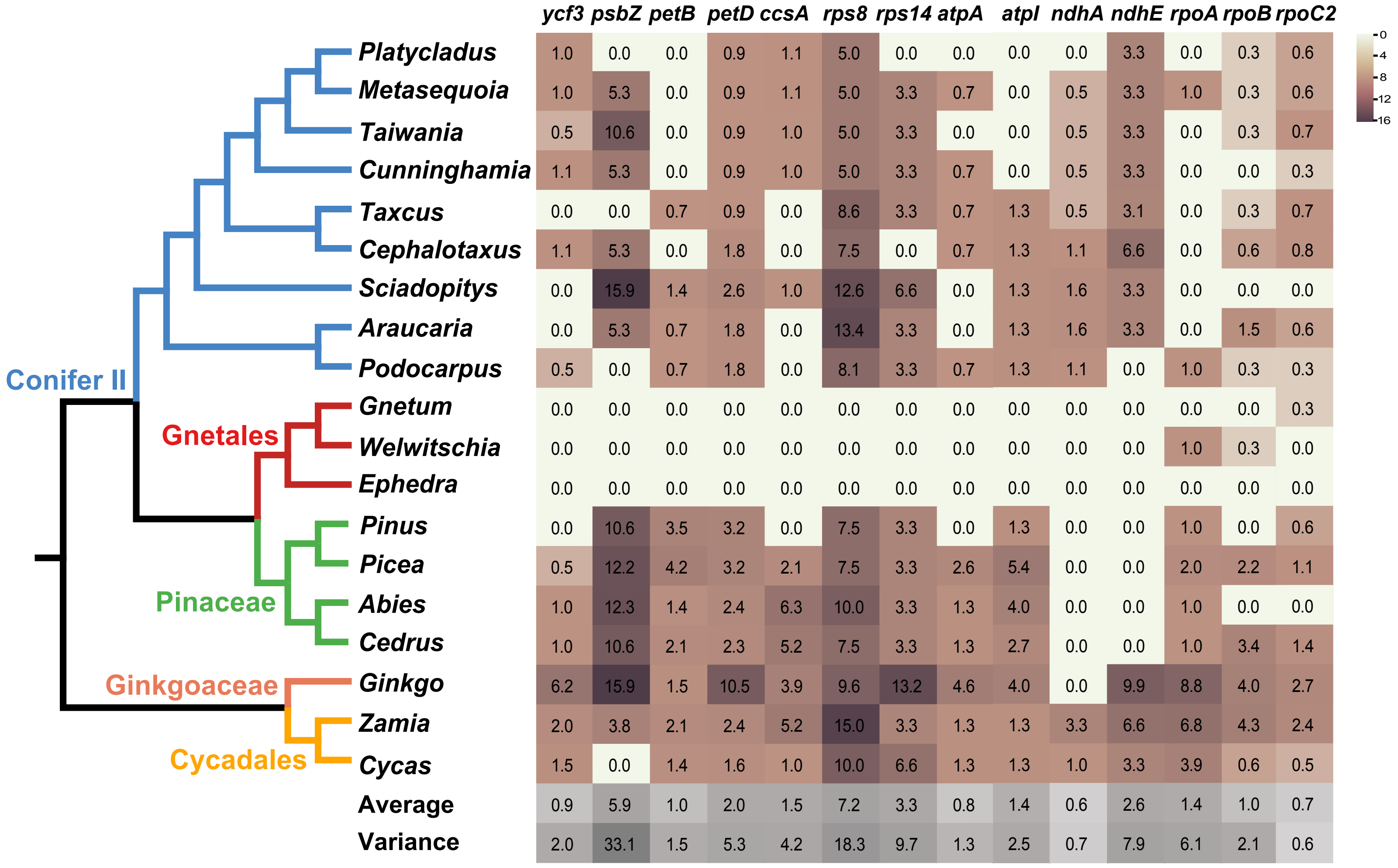

	ycf3	*psbZ*	*petB*	*petD*	*ccsA*	*rps8*	*rps14*	*atpA*	*atpI*	*ndhA*	*ndhE*	*rpoA*	*rpoB*	*rpoC2*
Platycladus	1.0	0.0	0.0	0.9	1.1	5.0	0.0	0.0	0.0	0.0	3.3	0.0	0.3	0.6
Metasequoia	1.0	5.3	0.0	0.9	1.1	5.0	3.3	0.7	0.0	0.5	3.3	1.0	0.3	0.6
Taiwania	0.5	10.6	0.0	0.9	1.0	5.0	3.3	0.0	0.0	0.5	3.3	0.0	0.3	0.7
Cunninghamia	1.1	5.3	0.0	0.9	1.0	5.0	3.3	0.7	0.0	0.5	3.3	0.0	0.0	0.3
Taxcus	0.0	0.0	0.7	0.9	0.0	8.6	3.3	0.7	1.3	0.5	3.1	0.0	0.3	0.7
Cephalotaxus	1.1	5.3	0.0	1.8	0.0	7.5	0.0	0.7	1.3	1.1	6.6	0.0	0.6	0.8
Sciadopitys	0.0	15.9	1.4	2.6	1.0	12.6	6.6	0.0	1.3	1.6	3.3	0.0	0.0	0.0
Araucaria	0.0	5.3	0.7	1.8	0.0	13.4	3.3	0.0	1.3	1.6	3.3	0.0	1.5	0.6
Podocarpus	0.5	0.0	0.7	1.8	0.0	8.1	3.3	0.7	1.3	1.1	0.0	1.0	0.3	0.3
Gnetum	0.0	0.0	0.0	0.0	0.0	0.0	0.0	0.0	0.0	0.0	0.0	0.0	0.0	0.3
Welwitschia	0.0	0.0	0.0	0.0	0.0	0.0	0.0	0.0	0.0	0.0	0.0	1.0	0.3	0.0
Ephedra	0.0	0.0	0.0	0.0	0.0	0.0	0.0	0.0	0.0	0.0	0.0	0.0	0.0	0.0
Pinus	0.0	10.6	3.5	3.2	0.0	7.5	3.3	0.0	1.3	0.0	0.0	1.0	0.0	0.6
Picea	0.5	12.2	4.2	3.2	2.1	7.5	3.3	2.6	5.4	0.0	0.0	2.0	2.2	1.1
Abies	1.0	12.3	1.4	2.4	6.3	10.0	3.3	1.3	4.0	0.0	0.0	1.0	0.0	0.0
Cedrus	1.0	10.6	2.1	2.3	5.2	7.5	3.3	1.3	2.7	0.0	0.0	1.0	3.4	1.4
Ginkgo	6.2	15.9	1.5	10.5	3.9	9.6	13.2	4.6	4.0	0.0	9.9	8.8	4.0	2.7
Zamia	2.0	3.8	2.1	2.4	5.2	15.0	3.3	1.3	1.3	3.3	6.6	6.8	4.3	2.4
Cycas	1.5	0.0	1.4	1.6	1.0	10.0	6.6	1.3	1.3	1.0	3.3	3.9	0.6	0.5
**Average**	0.9	5.9	1.0	2.0	1.5	7.2	3.3	0.8	1.4	0.6	2.6	1.4	1.0	0.7
**Variance**	2.0	33.1	1.5	5.3	4.2	18.3	9.7	1.3	2.5	0.7	7.9	6.1	2.1	0.6

Figure S4. Editing site densities of genes containing C-to-U RNA editing sites in at least ten species in 19 gymnosperm species. The editing site density is expressed as the number of editing sites per 1000 bp. Average editing site densities and their variances are shown in the bottom rows.
