# Supplementary material for: A Comprehensive Evolutionary Study of Chloroplast RNA Editing in Gymnosperms: A Novel Type of G-to-A RNA Editing Is Common in Gymnosperms
Source: Int J Mol Sci. 2022 Sep 16;23(18):10844. doi: 10.3390/ijms231810844 (PMC9505161; doi:10.3390/ijms231810844)
Supplement: Supplementary file 1 [file ijms-23-10844-s001.zip › fig S5.pdf]

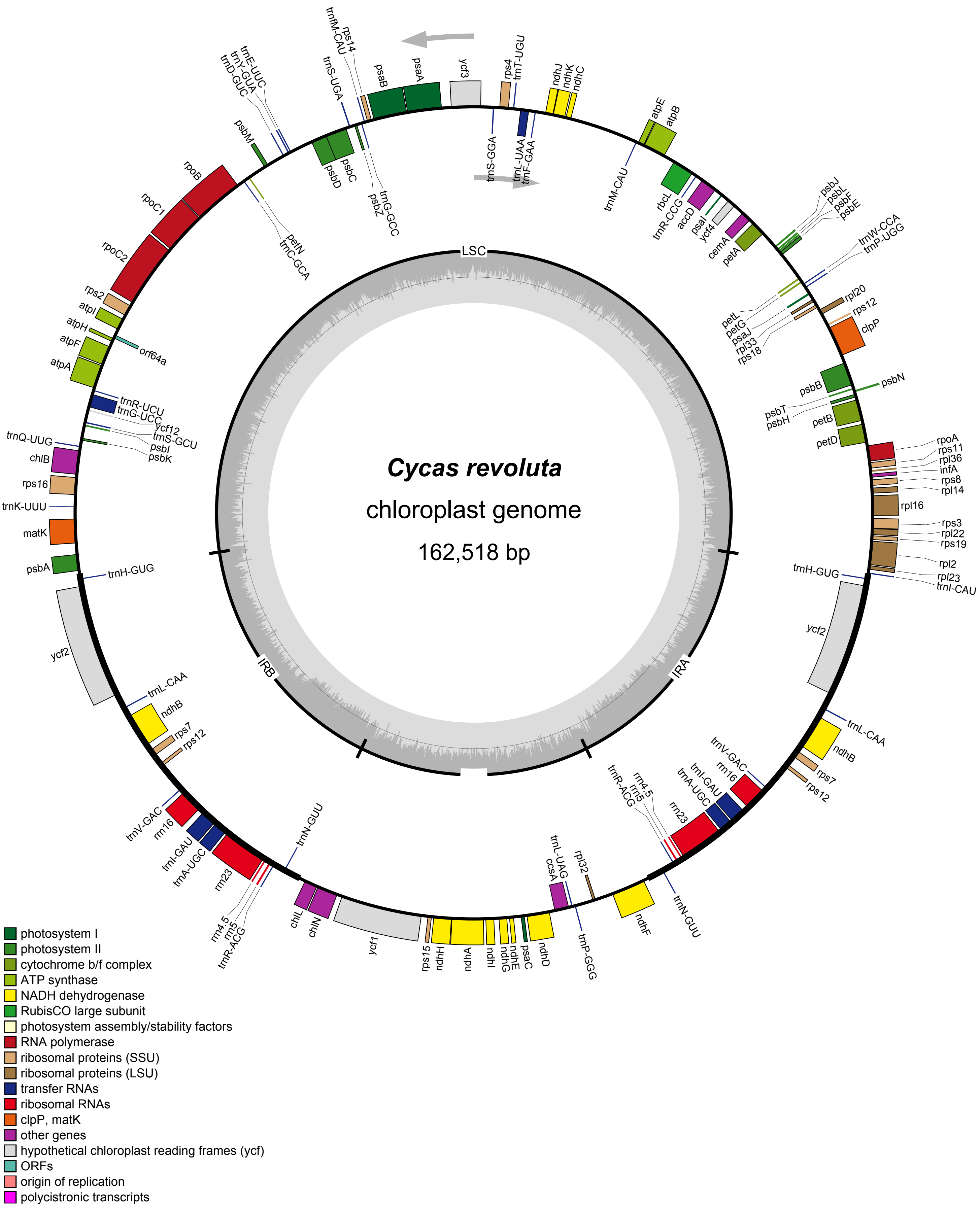

Figure S5. Map of the 19 gymnosperm plastomes drawn with the OGDRAW tool. Genes inside and outside the outer circle are transcribed clockwise and counter-clockwise, respectively. The darker grey in the inner circle represents the GC content and the lighter gray indicates the AT content. Genes belonging to different functional groups are clored-coded.

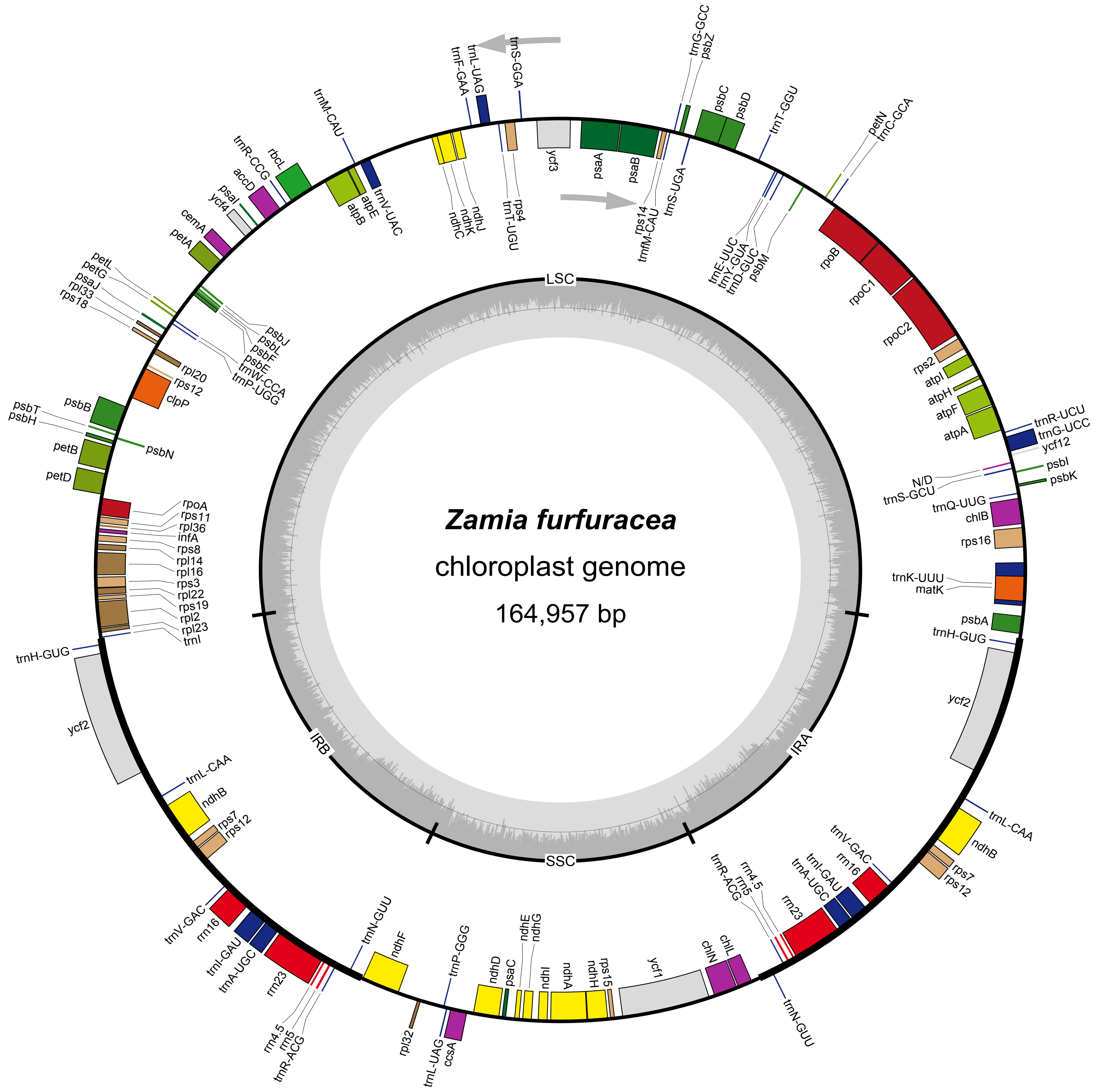

Figure S5 (continued)

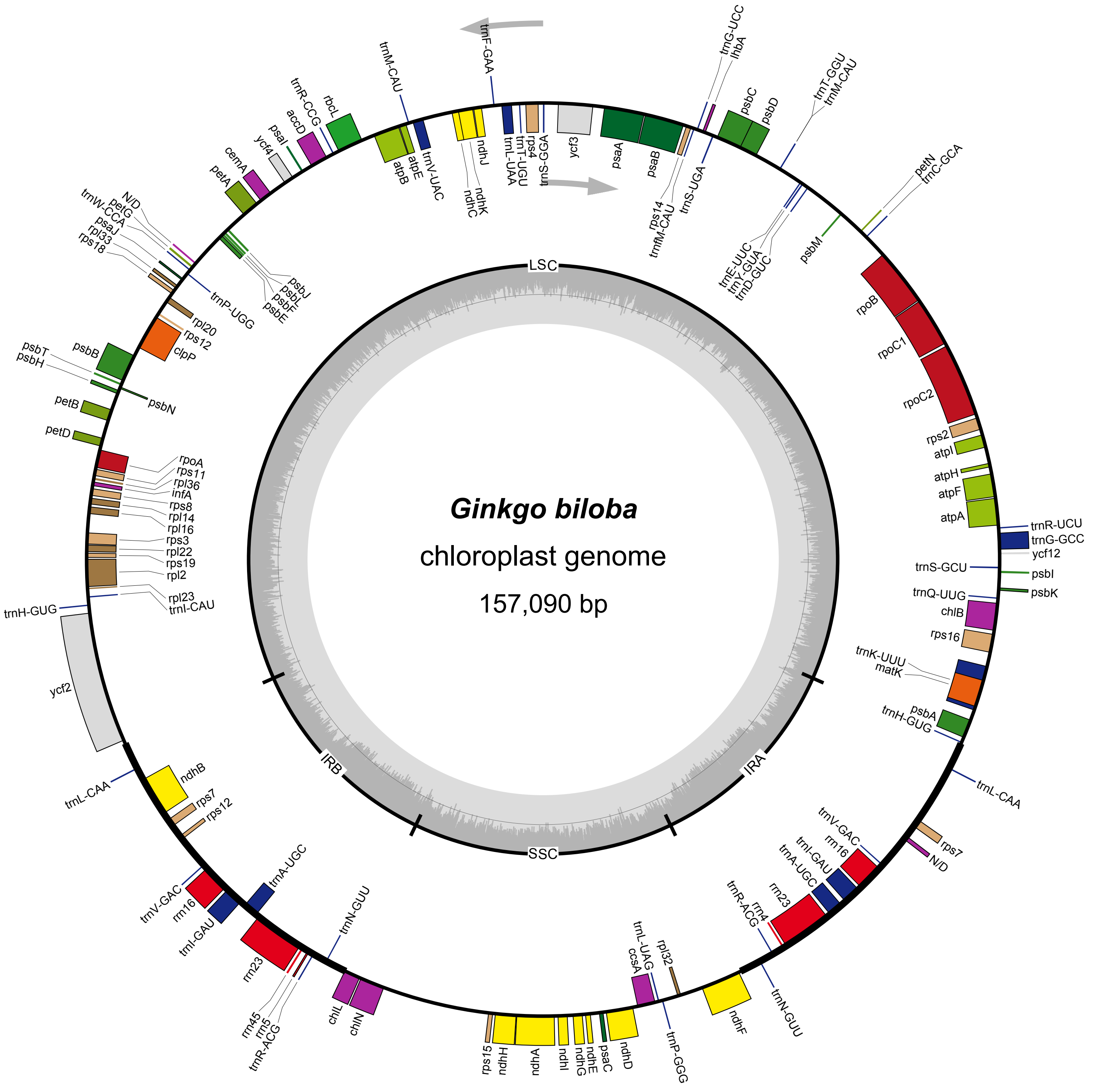

Figure S5 (continued)

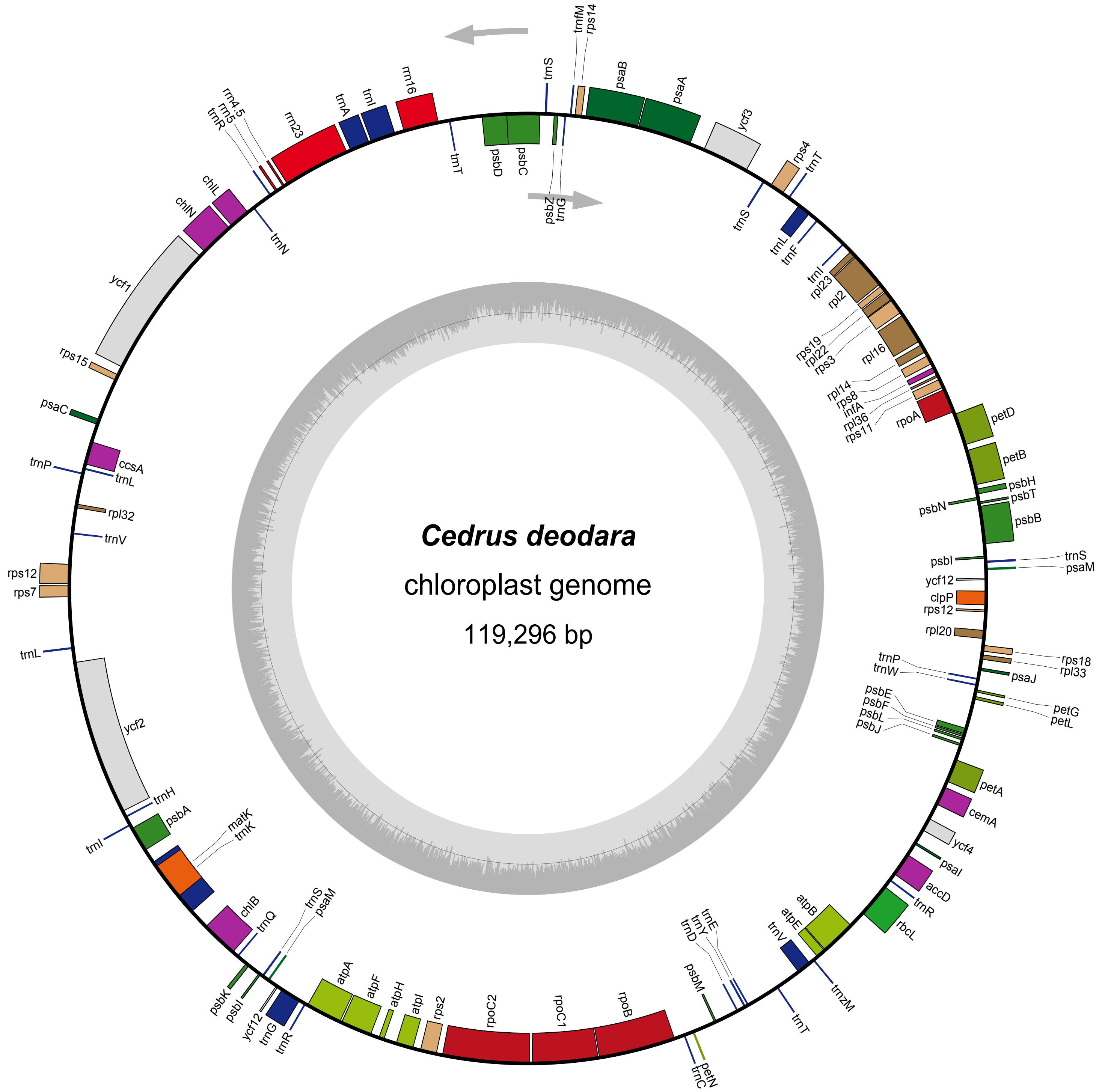

Figure S5 (continued)

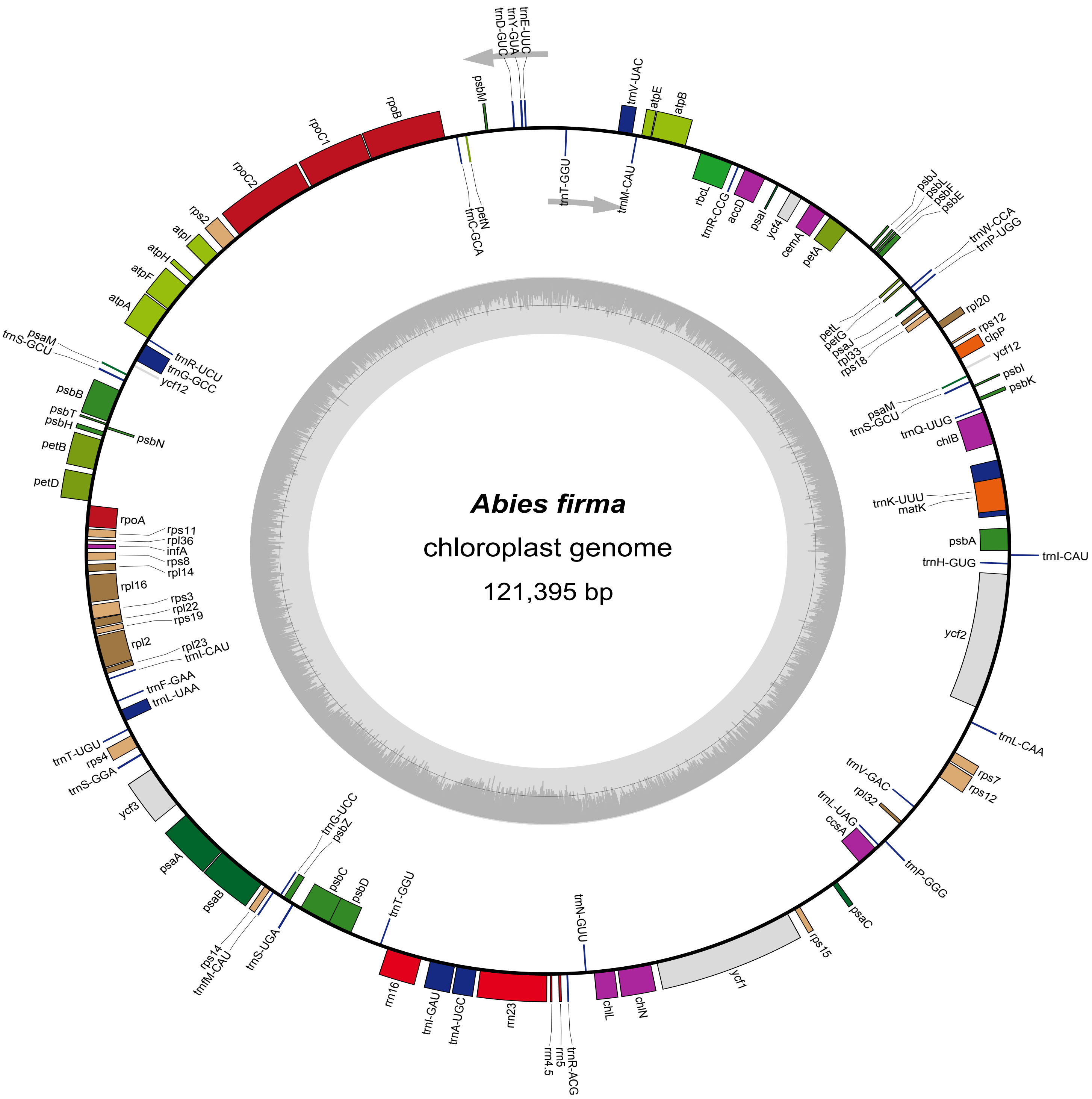

Figure S5 (continued)

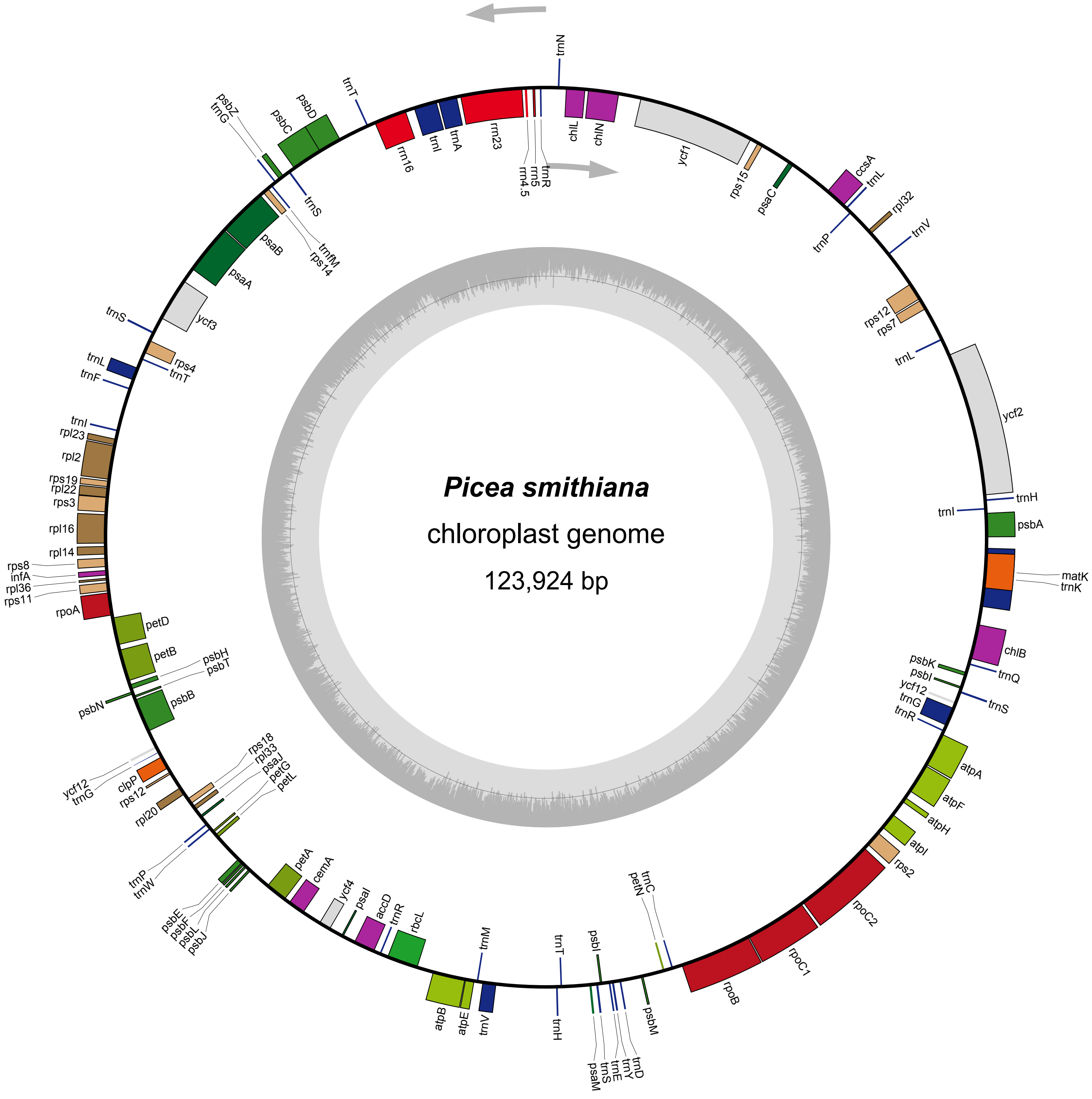

Figure S5 (continued)

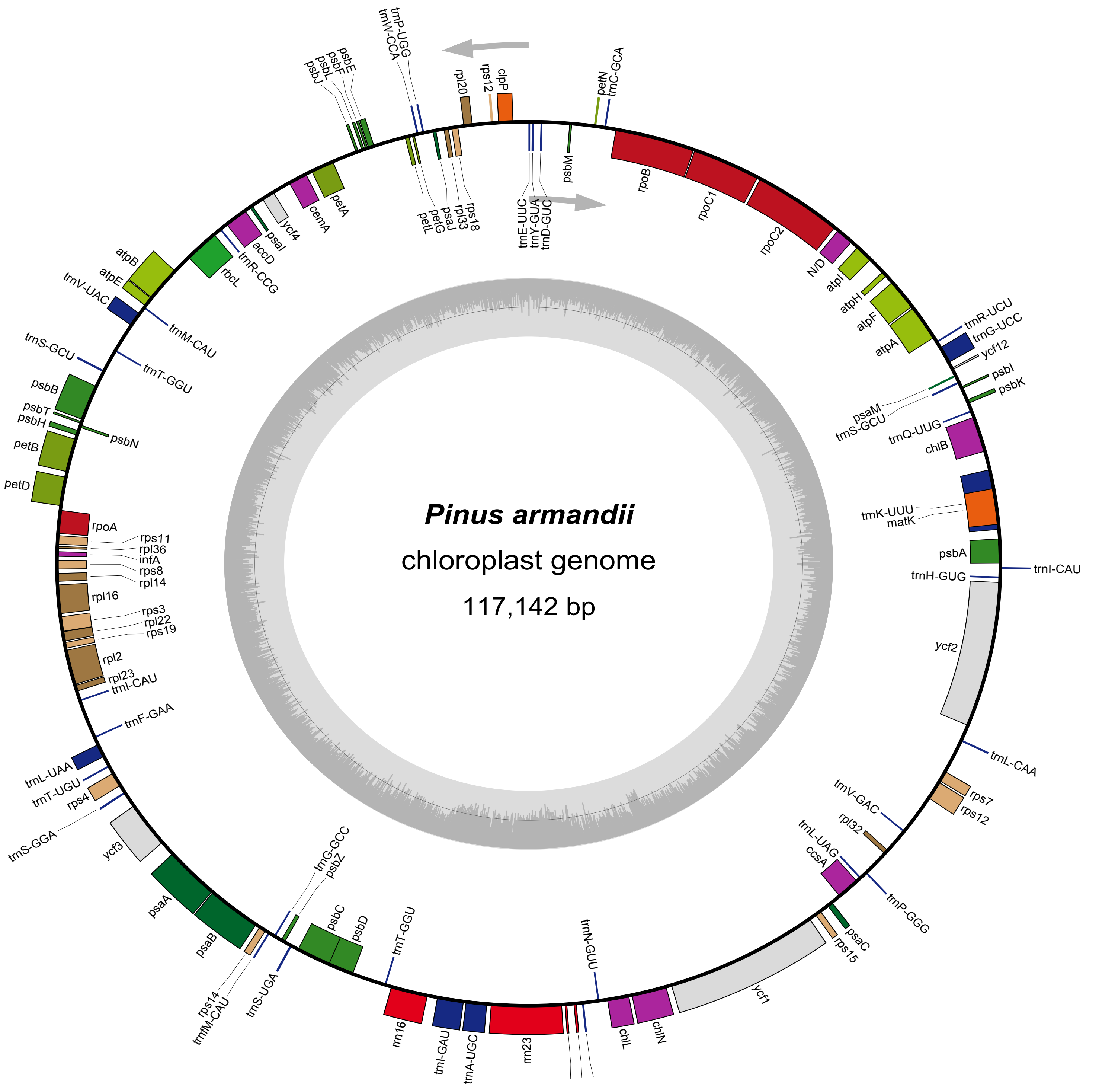

Figure S5 (continued)

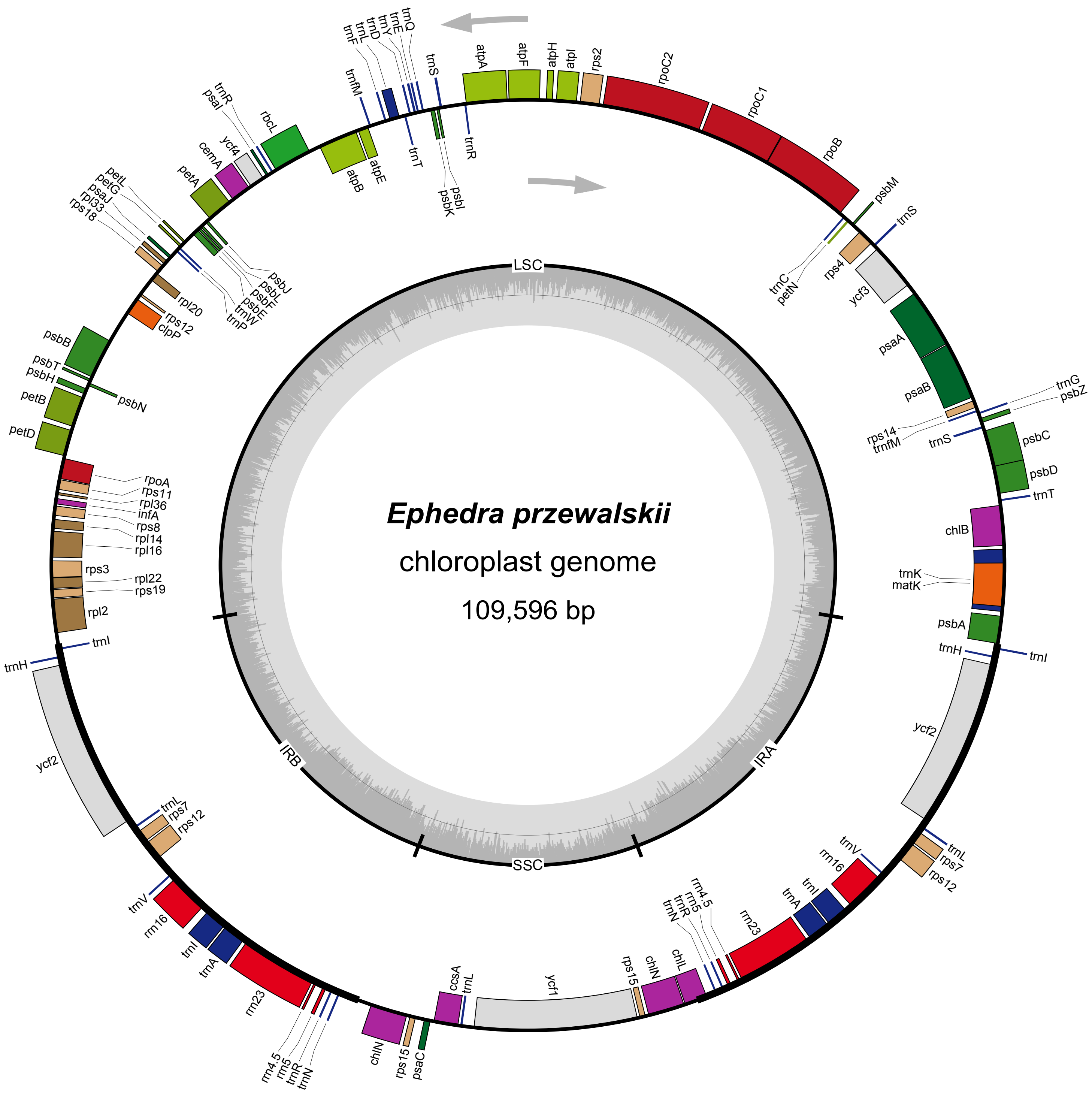

Figure S5 (continued)

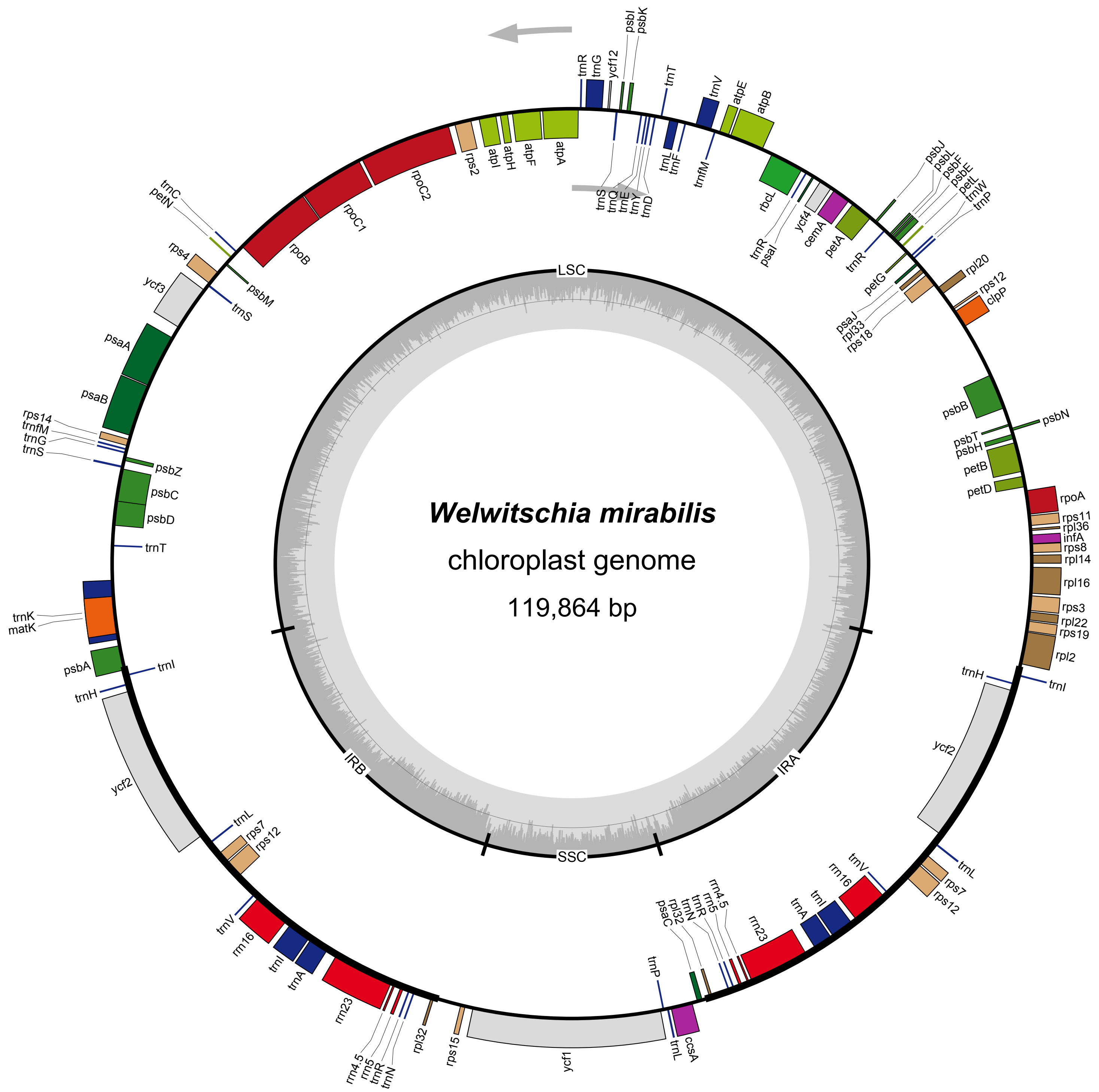

Figure S5 (continued)

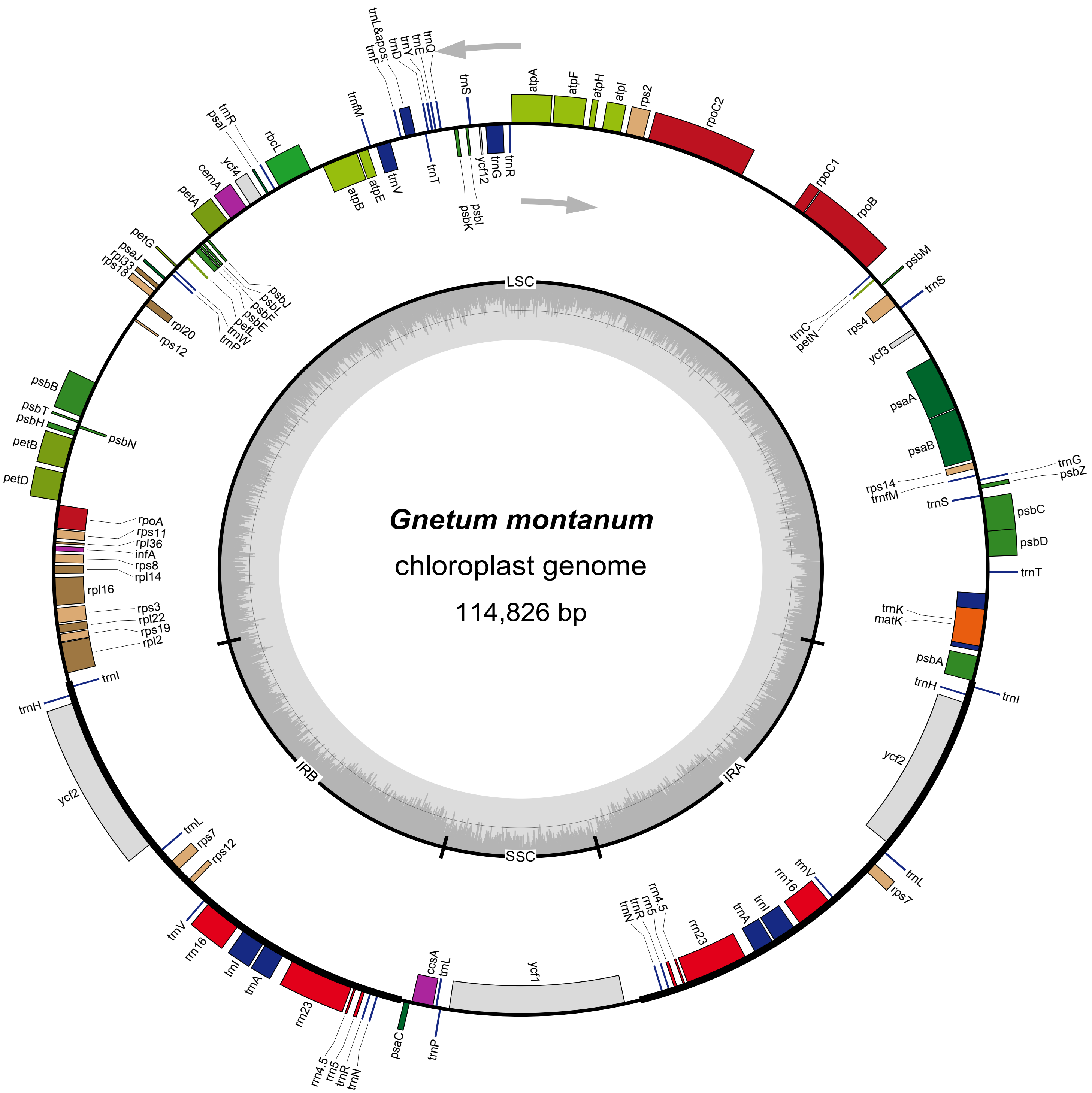

Figure S5 (continued)

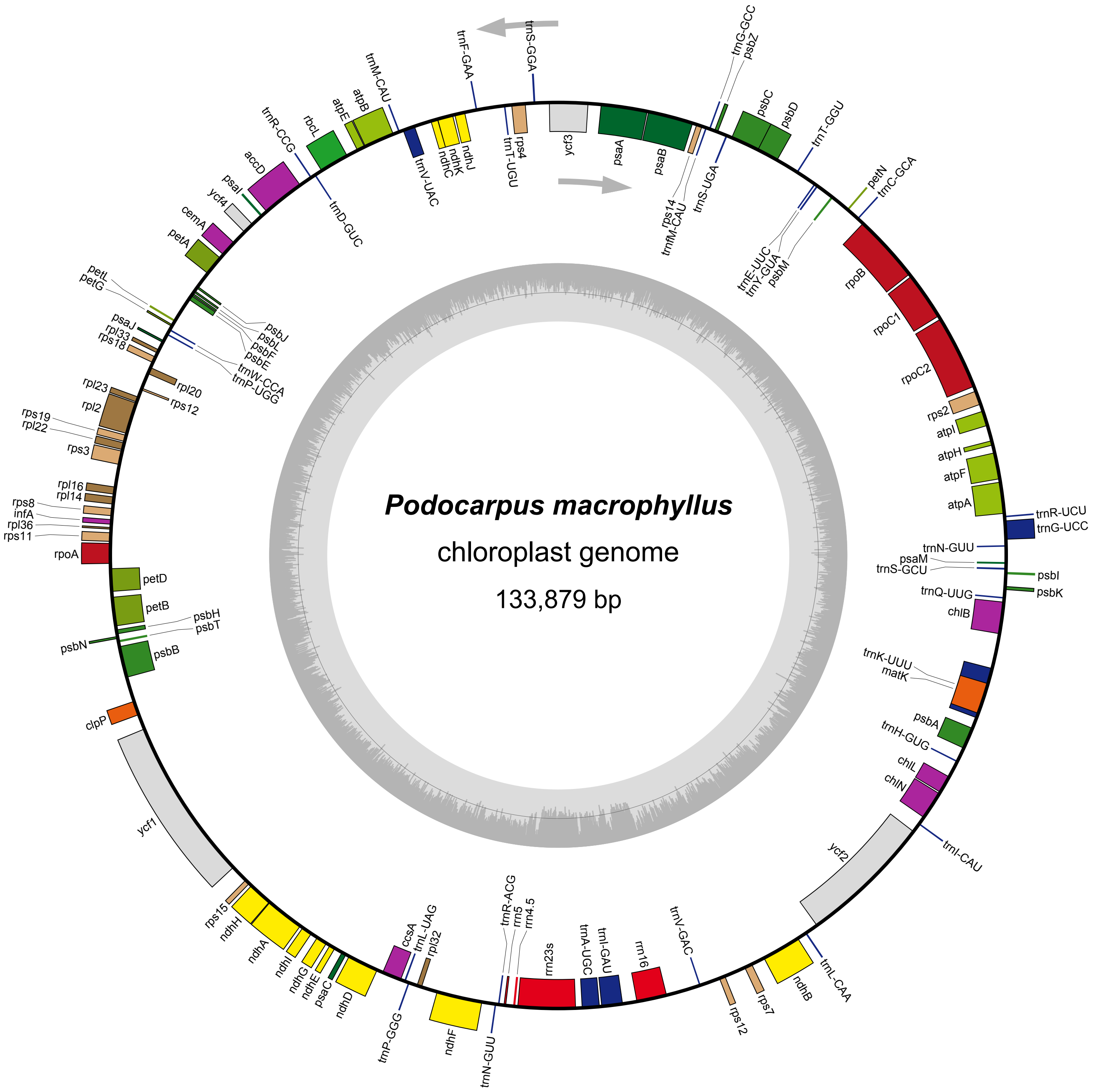

Figure S5 (continued)

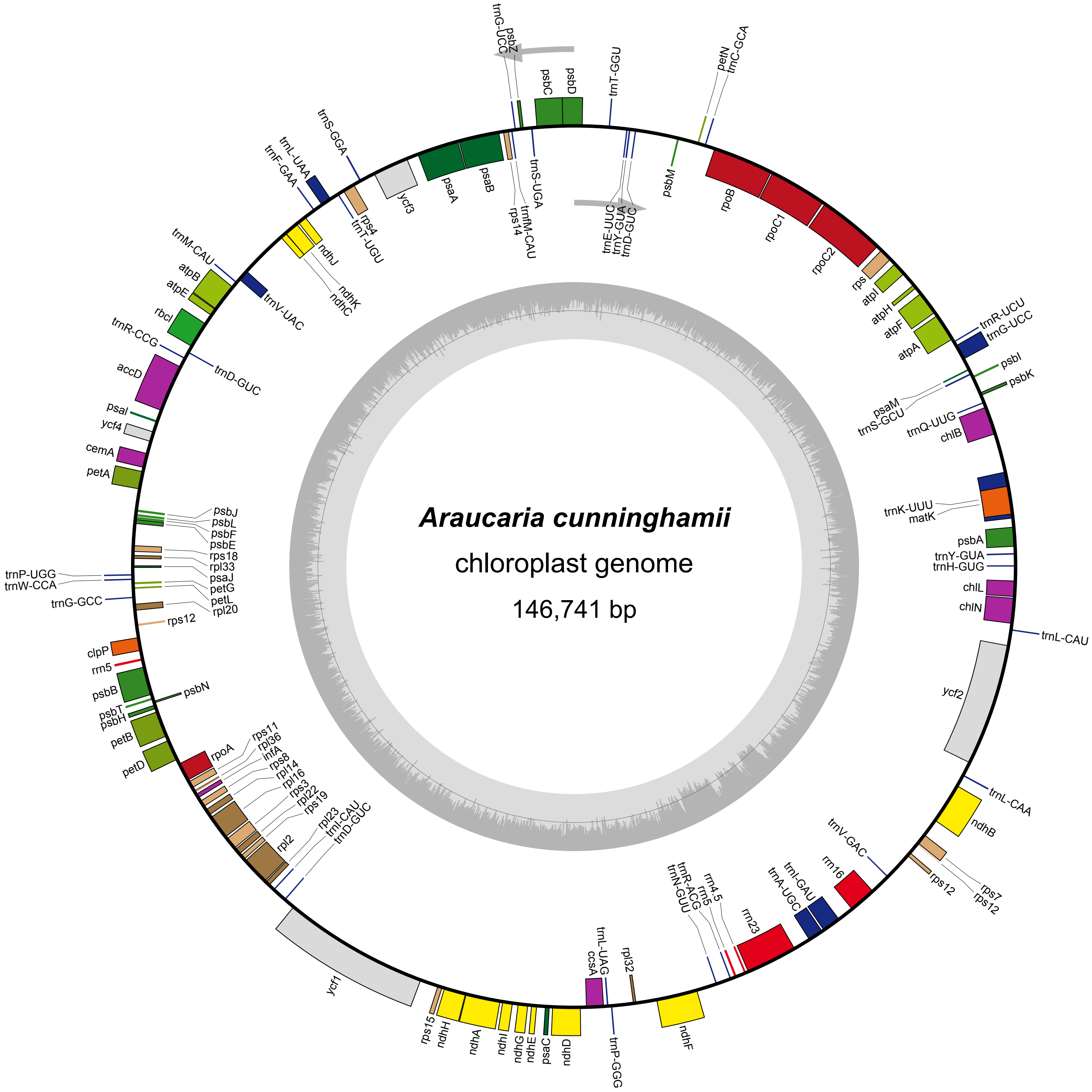

Figure S5 (continued)

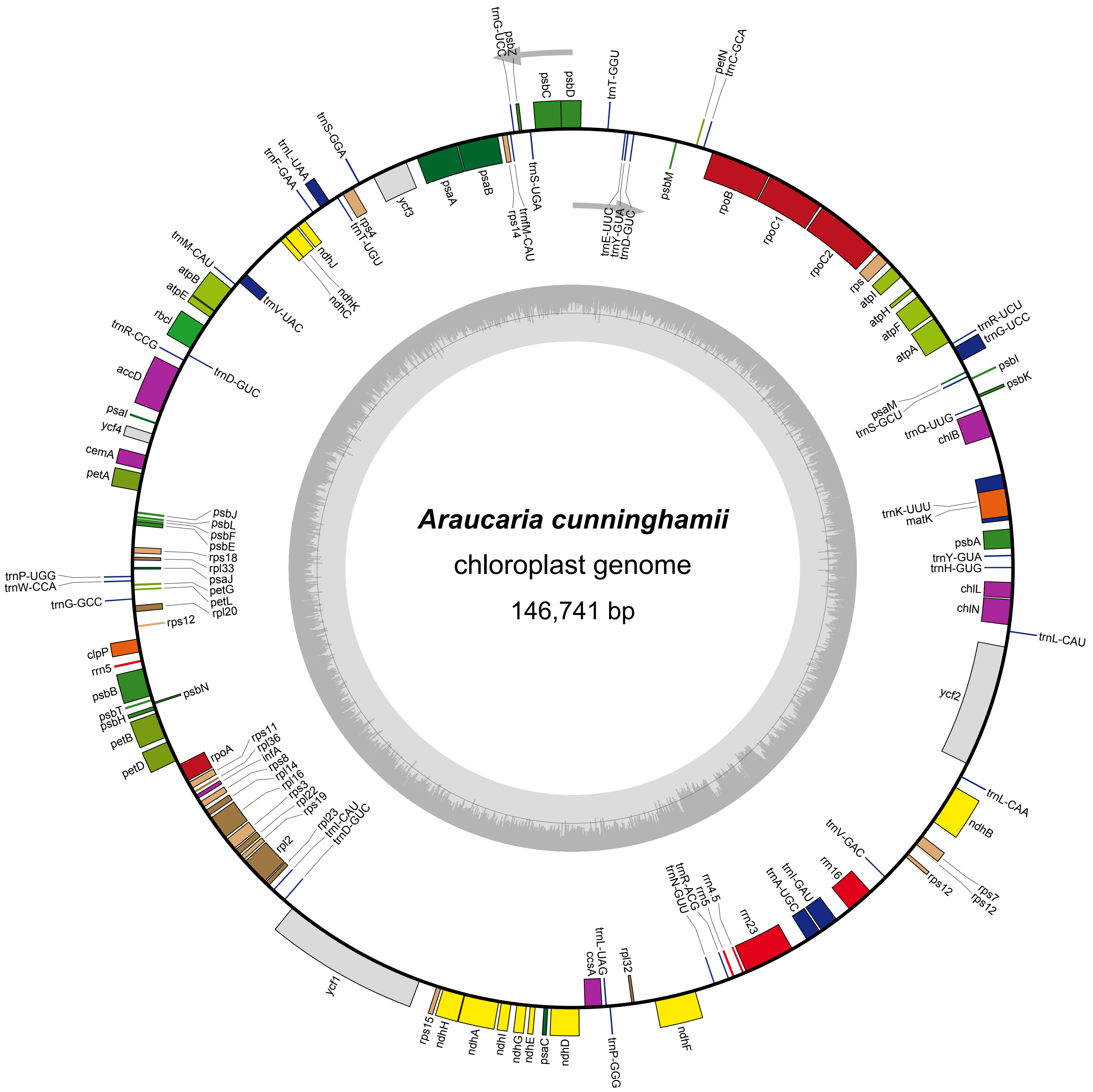

Figure S5 (continued)

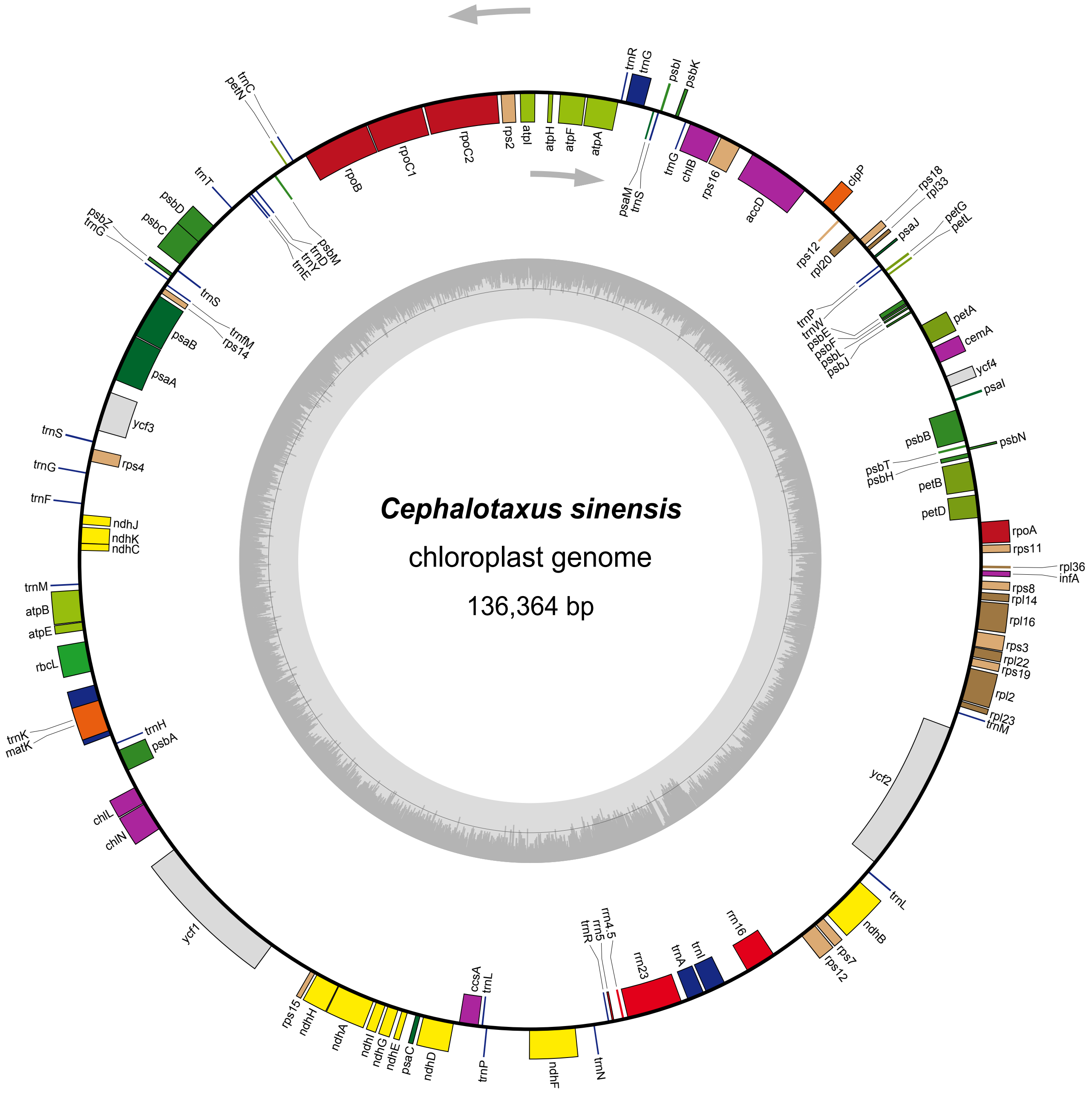

Figure S5 (continued)

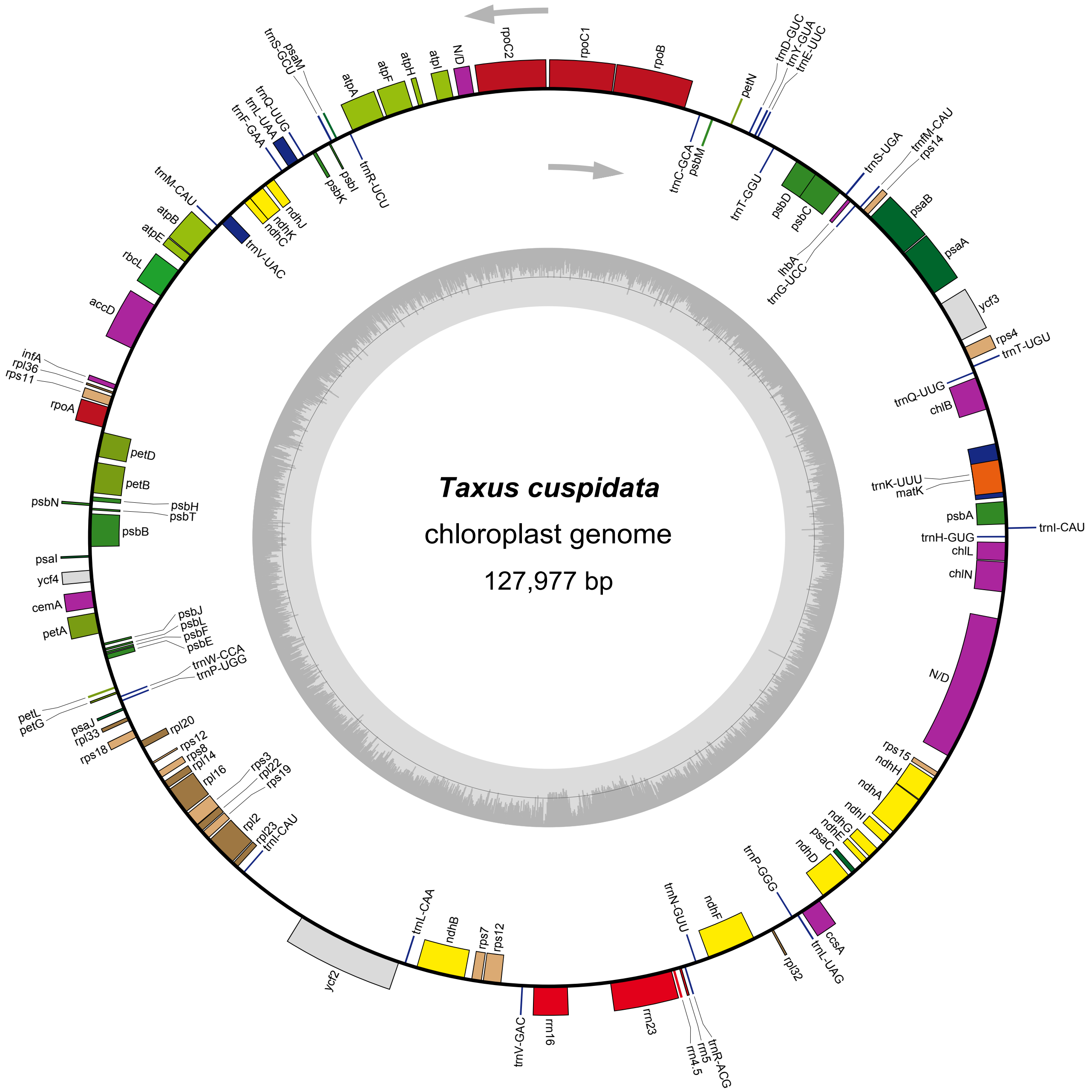

Figure S5 (continued)

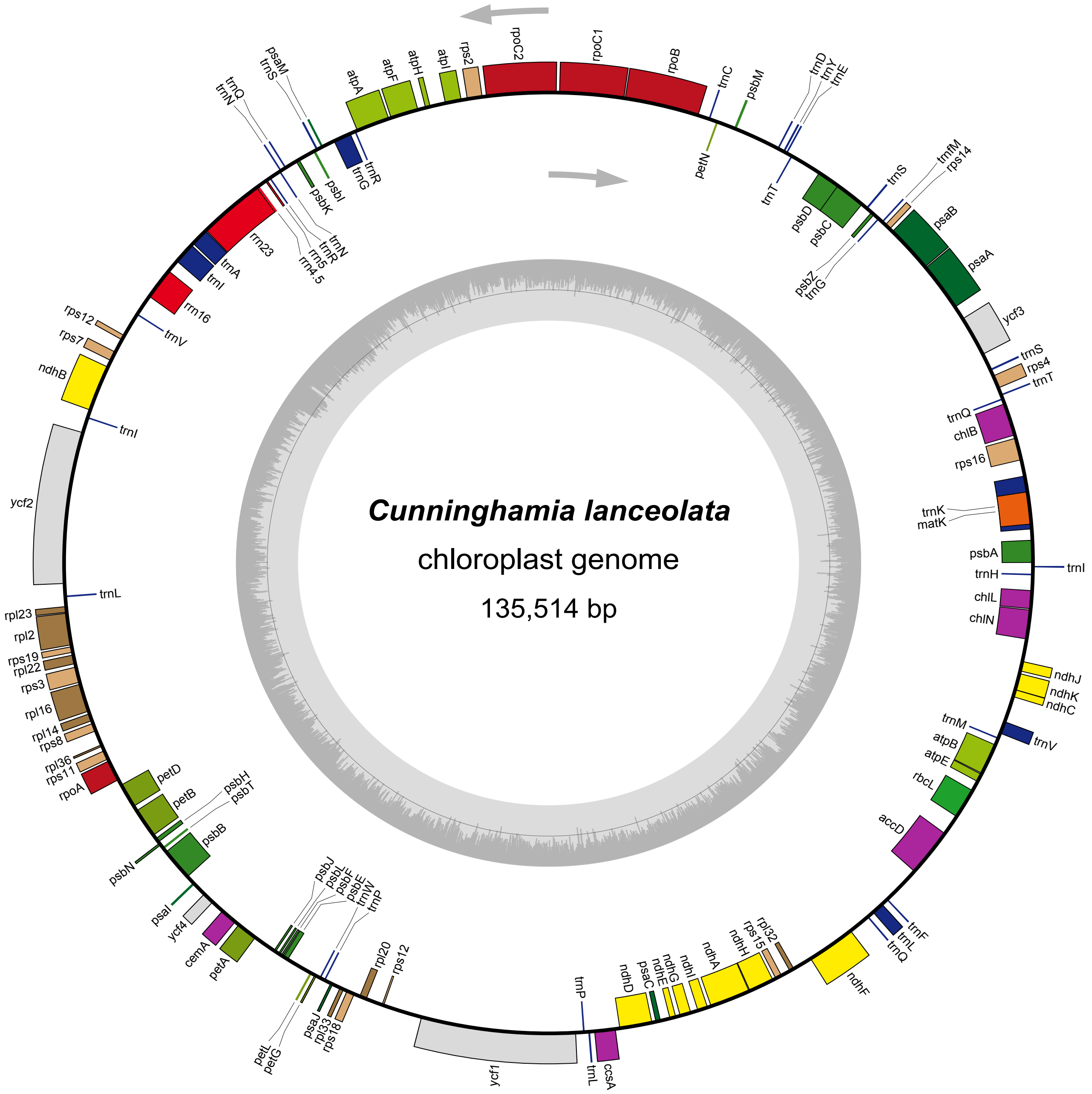

Figure S5 (continued)

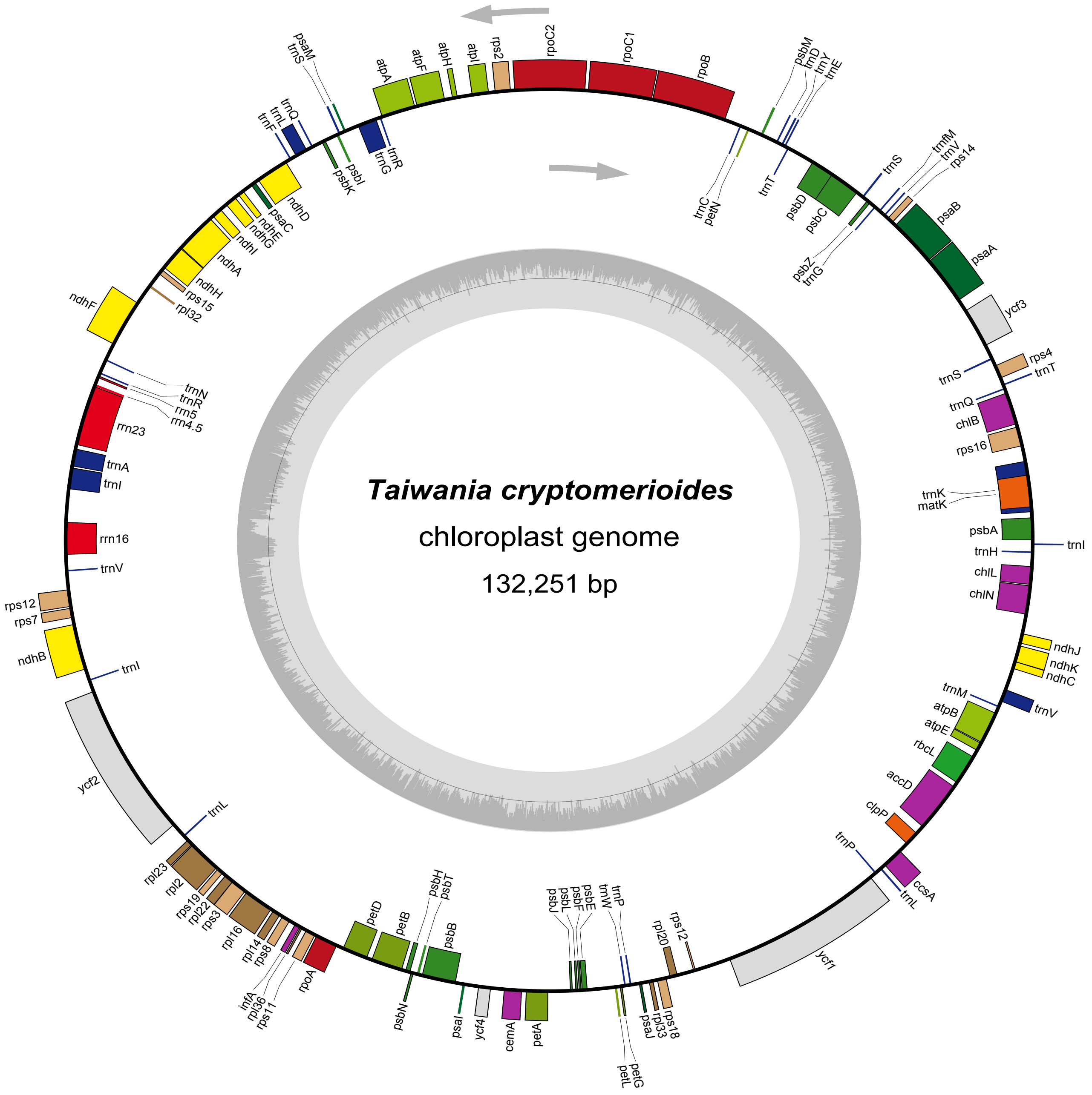

Figure S5 (continued)

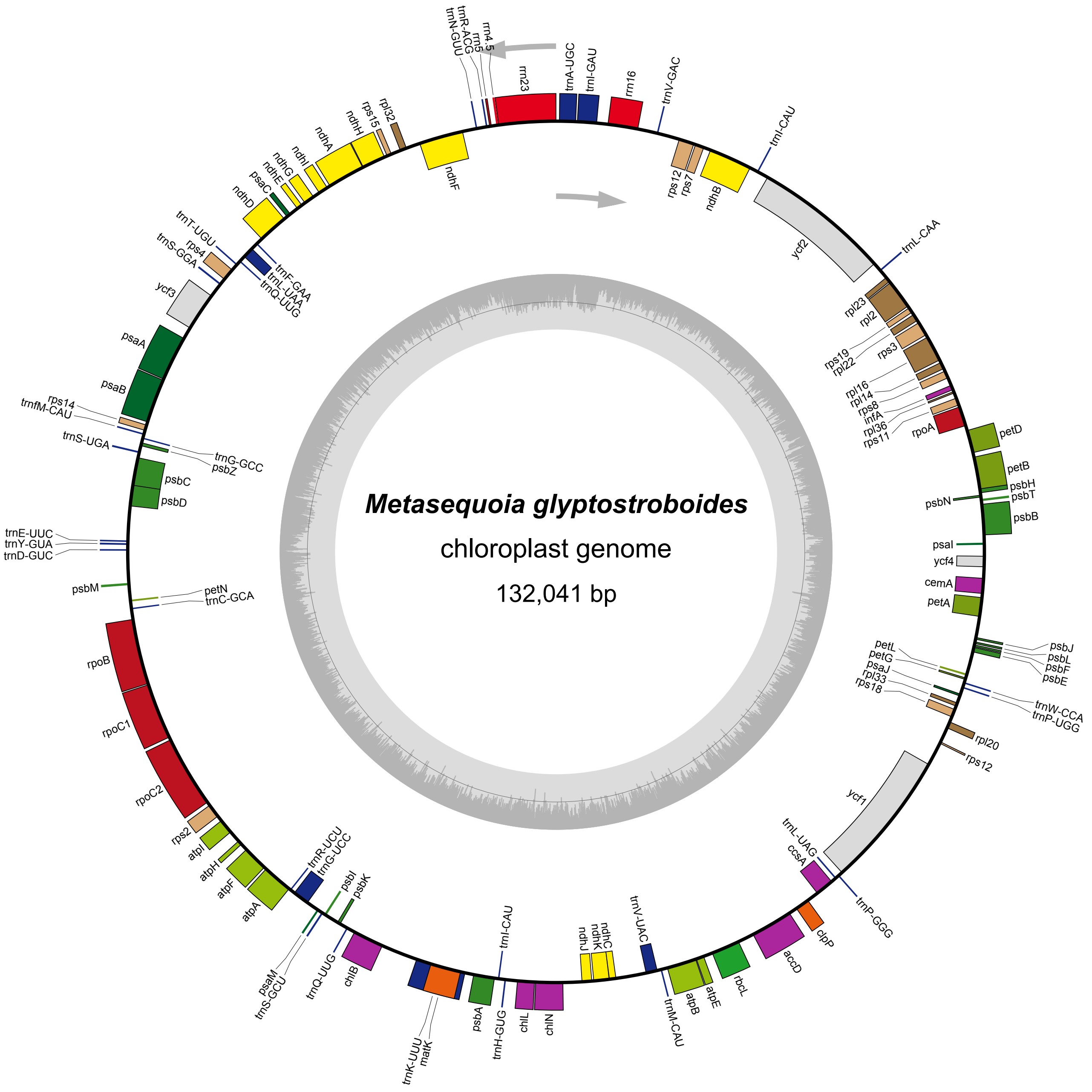

Figure S5 (continued)

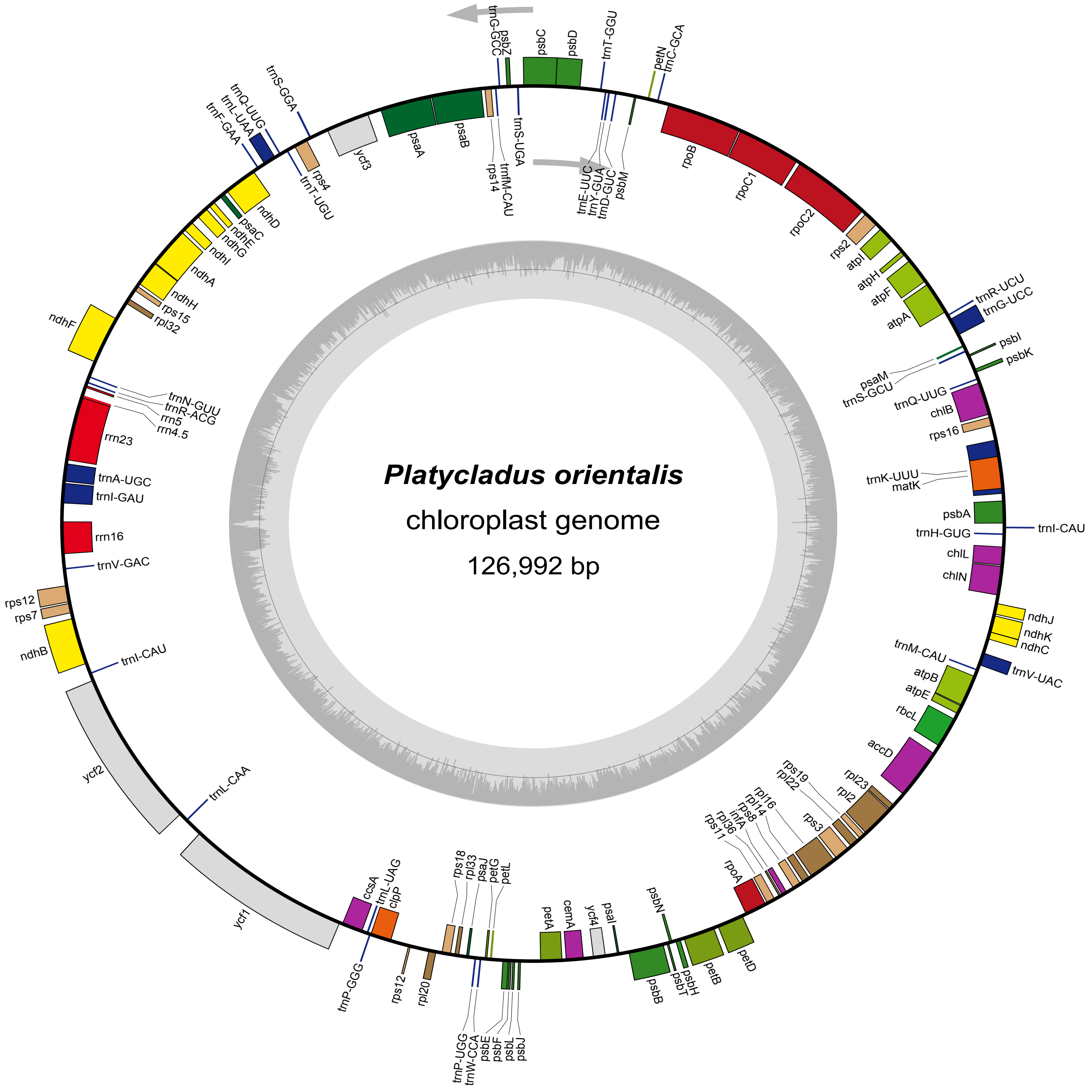

Figure S5 (continued)
