# Supplementary material for: A Comprehensive Evolutionary Study of Chloroplast RNA Editing in Gymnosperms: A Novel Type of G-to-A RNA Editing Is Common in Gymnosperms
Source: Int J Mol Sci. 2022 Sep 16;23(18):10844. doi: 10.3390/ijms231810844 (PMC9505161; doi:10.3390/ijms231810844)
Supplement: Supplementary file 1 [file ijms-23-10844-s001.zip › Table S4.pdf]

Table S4. G-to-A RNA editing sites in *rrn23* of the 16 gymnosperm species.

**Species**	**Position#**	**Editing efficiency**
*Cycas revoluta**	*rrn23*-3039	38.90%
*Cycas revoluta**	*rrn23*-3039	45.40%
*Zamia furfuracea**	*rrn23*-3039	36.10%
*Zamia furfuracea**	*rrn23*-3039	31.70%
*Ginkgo biloba**	*rrn23*-3039	54.00%
*Ginkgo biloba**	*rrn23*-3039	48.69%
Picea smithiana	*rrn23*-3039	25.90%
Abies firma	*rrn23*-3039	39.40%
Cedrus deodara	*rrn23*-3039	63.27%
Platycladus orientalis	*rrn23*-3039	50.20%
Metasequoia glyptostroboides	*rrn23*-3039	56.80%
Taiwania cryptomerioides	*rrn23*-3039	47.40%
Cunninghamia lanceolata	*rrn23*-3039	33.57%
Cephalotaxus sinensis	*rrn23*-3039	32.60%
Taxus cuspidata	*rrn23*-3039	53.29%
Sciadopitys verticillata	*rrn23*-3039	50.80%
Araucaria cunninghamii	*rrn23*-3039	36.43%
*Welwitschia mirabilis**	*rrn23*-3039	63.40%
*Welwitschia mirabilis**	*rrn23*-3039	55.30%
*Gnetum montanum**	*rrn23*-3039	57.69%
*Gnetum montanum**	*rrn23*-3039	49.85%

#The position is based on the alignment of the *rrn23* genes

* Both copies of *rrn23* have G-to-A RNA editing sites
