# Supplementary material for: A Comprehensive Evolutionary Study of Chloroplast RNA Editing in Gymnosperms: A Novel Type of G-to-A RNA Editing Is Common in Gymnosperms
Source: Int J Mol Sci. 2022 Sep 16;23(18):10844. doi: 10.3390/ijms231810844 (PMC9505161; doi:10.3390/ijms231810844)
Supplement: Supplementary file 1 [file ijms-23-10844-s001.zip › Table S5.pdf]

Table S5. Samples and data used in this study.

Family	Species	Abbr.	Sources	DNA-Seq		RNA-Seq		Accession number of chloroplast genomes
				SRA number	Sources	SRA number	Sources	
Cycadaceae	*Cycas revoluta*	*Cycas*	IBCAS	SRR12710844	Kan et al. [84]	SRX3059933	Ran et al. [27]	OM735459
Zamiaceae	*Zamia furfuracea*	*Zamia*	IBCAS	SRR12710843	Kan et al. [84]	SRX3059934	Ran et al. [27]	OM735446
Ginkgoaceae	*Ginkgo biloba*	*Ginkgo*	IBCAS	SRR12710832	Kan et al. [84]	SRX3059931	Ran et al. [27]	OM735452
Pinaceae	*Cedrus deodara*	*Cedrus*	IBCAS	SRR12710827	Kan et al. [84]	SRX3744356	Ran et al. [34]	OM735453
	Abies firma	*Abies*	IBCAS	SRR12710828	Kan et al. [84]	SRX3055044	Ran et al. [27]	OM735442
	Picea smithiana	*Picea*	IBCAS	SRR12710829	Kan et al. [84]	SRX3052146	Ran et al. [27]	OM735455
	Pinus armandii	*Pinus*	IBCAS	SRR12710830	Kan et al. [84]	SRX3057314	Ran et al. [27]	OM735456
Ephedraceae	*Ephedra przewalskii*	*Ephedra*	IBCAS	SRR12710836	Kan et al. [84]	SRR12710834	Kan et al. [84]	OM735444
Welwitschiaceae	*Welwitschia mirabilis*	*Welwitschia*	Buy from https://sheffields.com/	SRR12710831	Kan et al. [84]	SRX3059925	Ran et al. [27]	OM735458
Gnetaceae	*Gnetum montanum*	*Gnetum*	Hainan, China	SRR12710833	Kan et al. [84]	SRX3059920	Ran et al. [27]	OM735450
Podocarpaceae	*Podocarpus macrophyllus*	*Podocarpus*	IBCAS	SRR12710837	Kan et al. [84]	SRX3058149	Ran et al. [27]	OK094233
Araucariaceae	*Araucaria cunninghamii*	*Araucaria*	IBCAS	SRR12710838	Kan et al. [84]	SRX3057826	Ran et al. [27]	OM735457
Sciadopityaceae	*Sciadopitys verticillata*	*Sciadopitys*	KIBCAS	SRR12710839	Kan et al. [84]	SRX3058164	Ran et al. [27]	OM735447
Cephalotaxaceae	*Cephalotaxus sinensis*	*Cephalotaxus*	IBCAS	SRR12710840	Kan et al. [84]	SRX3057874	Ran et al. [27]	OM735448
Taxaceae	*Taxus cuspidata*	*Taxus*	IBCAS	SRR10305026	Kan et al. [85]	SRR10305025	Kan et al. [85]	OM735454
Cupressaceae	*Cunninghamia lanceolata*	*Cunninghamia*	KIBCAS	SRR12710824	Kan et al. [84]	SRX3058052	Ran et al. [27]	OM735443
	Taiwania cryptomerioides	*Taiwania*	KIBCAS	SRR12710842	Kan et al. [84]	SRR12710841	Kan et al. [84]	OM735445
	Metasequoia glyptostroboides	*Metasequoia*	IBCAS	SRR12710825	Kan et al. [84]	SRX3058056	Ran et al. [27]	OM735451
	Platycladus orientalis	*Platycladus*	IBCAS	SRR12710826	Kan et al. [84]	SRX3058062	Ran et al. [27]	OM735449

IBCAS: Institute of Botany, Chinese Academy of Sciences, Beijing, China; KIBCAS: Kunming Institute of Botany, Chinese Academy of Sciences, Yunnan, China.
