# Supplementary material for: A Comprehensive Evolutionary Study of Chloroplast RNA Editing in Gymnosperms: A Novel Type of G-to-A RNA Editing Is Common in Gymnosperms
Source: Int J Mol Sci. 2022 Sep 16;23(18):10844. doi: 10.3390/ijms231810844 (PMC9505161; doi:10.3390/ijms231810844)
Supplement: Supplementary file 1 [file ijms-23-10844-s001.zip › Table S6.pdf]

**Table S6.** Primers used in this study.

| Primer name | Primer Sequences（5' to 3'） |
|---|---|
| gap3_F1_Ara_cun- | TTGCGCATTACCGTTAATCCAT |
| gap3_R1_Ara_cun- | TGTACATACCAGATCACGAATTAGT |
| gap3_F2_Ara_cun- | TAGAATGGCAGAGGCCAATAGAGCT |
| gap3_F3_Ara_cun- | GCGCATTACCGTTAATCCATTCCAT |
| gap3_R2_Ara_cun- | GCAAGATTGTTATGCTCAGTAC |
| gap3_R3_Ara_cun- | GTACATTGAATGTATTCCACGG |
| gap1_F1_Cep_sin- | AAGGCCAACTGTGCCAACTGT |
| gap1_R1_Cep_sin- | CTTGCTCGAACGTAAATAACT |
| gap1_F2_Cep_sin- | AATCCAGGAAAATATGTGCC |
| gap2_F1_Cep_sin- | CATATCTTCTAGATAGGCCATGT |
| gap2_R1_Cep_sin- | TCTTGTGCTACAGAAGACGAATT |
| gap5_F1_Cep_sin- | TATAGAAGACTTGGATCGGACCACCT |
| gap5_R1_Cep_sin- | TACAAGAGCCTACAAGAGCCTAT |
| gap6_F1_Cep_sin- | TAGCTGGGATCACGTGAAATT |
| gap6_R1_Cep_sin- | GTTTGTACTTGAGGTGCCAAT |
| gap7_F1_Cep_sin- | TACTCCTTGTCAAACTTGATAGGT |
| gap7_R1_Cep_sin- | TTAACATAACAGCTGGCCATAT |
| gap8_F1_Cep_sin- | TATCCATGTGGCTAATAGCCTT |
| gap8_R1_Cep_sin- | CATAATTCATGGACGTTGATAAGGT |
| gap8_F2_Cep_sin- | TCAGGCGTTGATGAGCACATT |
| gap8_R2_Cep_sin- | GGAACAAGTGGATATGATCGATCCT |
| gap8_F3_Cep_sin- | TACGCCATGCTACTGTGCCTTGTAT |
| gap8_R3_Cep_sin- | TCGGCAGTGACTTACGTCACTT |
| gap8_F4_Cep_sin- | TCCATGTGGCTAATAGCCTTCACAGC |
| gap8_R4_Cep_sin- | TCGGCAGTGACTTACGTCACTTACGAT |
| gap8_F5_Cep_sin- | GAACAGAAGGAGTACGCCATGCTACTGT |
| gap2_F1_Cyc_rev- | TACGGAGATTAGCTGAACTAACTT |
| gap2_R1_Cyc_rev- | TCAGTAGAAAGATTACTGCCAAT |
| gap2_R2_Cyc_rev- | TCGATCTATCGATACAGAGAAT |
| gap5_F1_Eph_mer- | GGCATACAGCTTTCTGGATGT |
| gap5_F2_Eph_mer- | TAATACCGCGGATTGACCTTCT |
| gap5_R1_Eph_mer- | CCATTCAAGTTGAACGCCATAGT |
| gap6_F1_Eph_mer- | TTCCTAAGAGCAGCGTGTCTACCTAT |
| gap6_R1_Eph_mer- | TGCCGTCAATACAGCTACTGAGT |
| gap6_R2_Eph_mer- | AGAGCGTGATGATGACATGTAC |
| gap1_F1_Pin_arm- | GCAGTAGGAATTGCAAGGAACATT |
| gap1_R1_Pin_arm- | CGACATCGATATCTGTTCATATCGT |
| gap1_R2_Pin_arm- | TCATCGACCTAGCACTCGCATAAT |
| gap2_F1_Pin_arm- | TTCGTGAATCGCTAACGAACCT |
| gap2_R1_Pin_arm- | TGGAATGAATCAGACCGAGCTT |
| gap3_F1_Pin_arm- | TGATCCTGTAATTAGGAAATCGTCCT |

| Primer name | Primer Sequences（5’ to 3’） |
|---|---|
| gap3_F2_Pin_arm- | TAGCAGTTCCAATGCTACGCCTT |
| gap5_F1_Pin_arm- | TCCGTATAGAACGTAATTCACCT |
| gap5_R2_Pin_arm- | CGATATATCGTATCGGCTGAT |
| gap8_F1_Pin_arm- | TATCGATCGGACATGTGGCTT |
| gap8_R1_Pin_arm- | TCTACTCAAGAGCGAAGCTAT |
| gap8_R2_Pin_arm- | TAGGAAGTCAGTCTCTAGGTT |
| gap9_F1_Pin_arm- | GGATTTGGTCCATGGATTGCAT |
| gap9_R1_Pin_arm- | GCTAGATCGGATCAAACATGCT |
| gap9_F2_Pin_arm- | GCTCTATGAATCGATTGTAAGAT |
| gap1_F1_Pla_ori- | TAGAGTCCAATTCTACATGTCAATGT |
| gap1_R1_Pla_ori- | CTGTGTATCATACTAGCACAGT |
| gap1_R2_Pla_ori- | GTGTATATGCCAGGAACCAGATT |
| gap3_F1_Pla_ori- | TTATTCGATCCCGAATCGACATT |
| gap3_R1_Pla_ori- | TAGCAAACTTTGAACCGACTTCTACT |
| gap1_F1_Pod_mac- | TGTCCAGACTATTGGACATGTCT |
| gap1_R1_Pod_mac- | TCCTGACGGAACGAATCACACT |
| gap1_F2_Pod_mac- | TATGTACTTCGGACTGTTCTCT |
| gap2_F1_Pod_mac- | TCTGTTACTGGTTGTCCACCGAT |
| gap2_R1_Pod_mac- | TGGTCCGTTCCAATATCAGGAT |
| gap3_F1_Pod_mac- | GTTAATTGCTTCCACTGATCGT |
| gap3_R1_Pod_mac- | TTGTGCAATATGCCGACGCTGGTT |
| gap3_F2_Pod_mac- | GTATCGGCTAATAGCTGTTAT |
| gap3_R2_Pod_mac- | TGCATCAGGATAAATGGAATT |
| gap4_F1_Pod_mac- | TTGAACCAAGCCTCCGCAATT |
| gap4_R1_Pod_mac- | CAGTGGTGTAGCATCAGATCCT |
| gap5_F1_Pod_mac- | CTATTGCTGAACCTAAAGCGT |
| gap5_R1_Pod_mac- | TATAAGACCGAATGAGCGATAGGT |
| gap6_F1_Pod_mac- | GCAGAAGTGGAACTAAGATACCT |
| gap6_R1_Pod_mac- | CAATCTCGTCGTCAAGTGGCTT |
| gap6_F2_Pod_mac- | GTCCAGGATGTGTACAGAGAAT |
| gap6_R2_Pod_mac- | GCTCTTGAGTTACATCATCAAGACT |
| gap6_R3_Pod_mac- | GAGAACCACTGAAAATTACTTGGCT |
| gap6_R4_Pod_mac- | AATAGCGCACACGATTGGTGCAGCGAT |
| gap7_F1_Pod_mac- | GAGCTCAATTCCTATTACTCCGAAT |
| gap7_R1_Pod_mac- | CTCAGAGGTACTTCATAATCAGCTT |
| gap7_F2_Pod_mac- | TCTGAATGAAGTAGCAGAGCT |
| gap7_R2_Pod_mac- | GGTGTTCTAGTTCTGGATACT |
| gap9_F1_Pod_mac- | CATGTCTGCTATCTCTAGGAGGT |
| gap9_R1_Pod_mac- | TATGCCTTGAAGAGGACTCGAACCT |
| gap8_F1_Pod_mac- | GCACTGATTGCTGATACGAGAT |
| gap8_R1_Pod_mac- | TAGCTCAGAGGATTAGAGCACGT |
| gap8_R2_Pod_mac- | TCTACTGCGGTGACGATACTGT |
| gap8_F3_Pod_mac- | TACAGTATCGTCACCGCAGTAGAGT |

| Primer name | Primer Sequences（5’ to 3’） |
|---|---|
| gap8_R3_Pod_mac- | TAAGGTCACGGTGAGACTAGCCGTT |
| gap8_F4_Pod_mac- | TCAGTTCACCAGGTTGTCTCTT |
| gap8_R4_Pod_mac- | TACGACTACGATAGGTGTCAAGT |
| gap8_F5_Pod_mac- | GAACTATCTATGTGGCTGATAGCCT |
| gap8_R5_Pod_mac- | TCTATCGTTGGTTCCGAAGGT |
| gap8_R6_Pod_mac- | CTGCAAGACCAATAATGCAGATCT |
| gap8_F7_Pod_mac- | TCTCAGACCAGCTACTGATCGT |
| gap8_R7_Pod_mac- | TCATCGGTTCAACGATGAGCACT |
| gap8_F8_Pod_mac- | TGATCGTAGCCTTGGTAAGCCATT |
| gap8_R8_Pod_mac- | TATCTGAAGCAGGTCGCTCAAT |
| gap8_F9_Pod_mac- | AGCAATACAAGCCGCCTAGTGT |
| gap8_R9_Pod_mac- | TCACCTTGACGTGGTGATAGTCAT |
| gap8_F10_Pod_mac- | TCCAGGTGACACTCTACCACTGAGTT |
| gap8_R10_Pod_mac- | TTGACACTAGGCGGCTTGTATT |
| gap8_F11_Pod_mac- | TGTGAAGTTATCCTATCGCATG |
| gap8_R11_Pod_mac- | TTCCTGACAAGCAAGGTTGAACCT |
| gap8_F12_Pod_mac- | CATGAACGGAAGTTACATAACTTC |
| gap8_R12_Pod_mac- | TGACATGAACATGATAGGTGAGCT |
| gap8_R13_Pod_mac- | TAACTCAGTGGTAGAGTGTCACCT |
| gap9_F2_Pod_mac- | GATGGCAGGCAGGTTCCTATT |
| gap9_R2_Pod_mac- | TCAGGAATTATTGAGCAGCAGCGAT |
| gap9_F3_Pod_mac- | GAGGTATTCCTCCATTGGCAGGTT |
| gap9_R3_Pod_mac- | CGTACTCTCAGCCTATTGAGAGAAT |
| gap11_F1_Pod_mac- | GACTCAGTCAGATAGGACTTGTT |
| gap11_R1_Pod_mac- | TAACCATTCAGCCATGGATGCTAGT |
| gap11_F2_Pod_mac- | GTAGATCGCGTAGAATTAACGT |
| gap11_R2_Pod_mac- | TAGCTATCACTGGAATGGCTCATGCT |
| gap11_F3_Pod_mac- | TAGCATCCATGGCTGAATGGTT |
| gap11_R3_Pod_mac- | AGGCAGCGATCGGATCGAAT |
| gap11_F4_Pod_mac- | CAACTGGTCGTGGATTTACCGAT |
| gap11_R4_Pod_mac- | TGACCGTAACCAGAGAATGT |
| gap11_F5_Pod_mac- | TGACCACGTCTGGCTAGAGCAATT |
| gap11_R5_Pod_mac- | CAACATGTGTGAGTTCATGGT |
| gap11_F6_Pod_mac- | GAATTGCGGTCATGGTTAGAACT |
| gap11_R6_Pod_mac- | TGCTTCTTGAGCAATTGCCAGGAT |
| gap11_F7_Pod_mac- | GCCGAGTACTCTACCATTGAGTT |
| gap11_R7_Pod_mac- | TACTGCTAAATACGCTCTGTTGT |
| gap11_F8_Pod_mac- | TGAAGGAACTAGCTACTCGTAATGT |
| gap11_R8_Pod_mac- | CTGTTGAAGTAATGTATGCGGCT |
| gap11_R9_Pod_mac- | CGGCATCGTCTAGAAGAATATT |
| gap11_F10_Pod_mac- | GCGTGCATTAAGAGCATATACCT |
| gap11_R10_Pod_mac- | TTGCGAGATACATGCACACGGAT |
| gap11_R11_Pod_mac- | TCAAGCTGCTGAATTAGCACT |

| Primer name | Primer Sequences（5’ to 3’） |
|---|---|
| gap2_F1_Sci_ver- | TAGTAGAGGTGCCTACTACCTT |
| gap2_R1_Sci_ver- | CAGGTCGGAACAAGTTGATTGCAAAT |
| gap2_R2_Sci_ver- | AGTTCATGAGATAGCTCATTAT |
| gap1_F1_Tai_cry- | TGAACTATCTATGTGGCTGATAGCT |
| gap1_R1_Tai_cry- | TGAAGTTCATAAGTCAGCTCAT |
| gap2_F1_Tai_cry- | TCCGACTTGCATGTGTTAAGCAT |
| gap2_R1_Tai_cry- | TTGACGTGGTGAAAGCCATCAGTT |
| gap1_F1_Tax_cus- | TGTGCTACTCAACATTCTGGAT |
| gap3_F1_Tax_cus- | TATCTTTCGGCATTGTCGACCAAGT |
| gap3_R1_Tax_cus- | GTGATACTCCTTATGCAATGTACGAT |
| gap5_F1_Tax_cus- | TGCAGTGTATAGATCTGCATTATCACT |
| gap5_R1_Tax_cus- | GAATTTCGTTCGGATTCAGAGGATAT |
| gap3_F4_Ara_cun* | TGCGCATTACCGTTAATCCATTCCAT |
| gap3_R4_Ara_cun* | TGTATTCCACGGATAGAGGAAT |
| gap3_F5_Ara_cun* | TTAATAAGTTCTCTCGGTCGG |
| gap3_R5_Ara_cun* | GCTGATCATAGGTTCTTCTCTCCATCG |
| gap8_F2_Pin_arm* | CCGAACAACTTGTTCGAGAGTT |
| gap8_F3_Pin_arm* | TATCGGACTTGAACCGATGACT |
| gap8_R3_Pin_arm* | TGAGAGCTAGAATACTTGCTGTGCT |
| gap8_R4_Pin_arm* | CGCGTAGGTTACTACTAATCG |
| gap1_F2_Tai_cry* | TTGTCACCTCTTATAATTAG |
| gap1_R2_Tai_cry* | TCGAGACAAGTTGATAGATTACAT |
| gap1_F3_Tai_cry* | GTAGTTAAACCAATCAATGTT |
| gap1_R3_Tai_cry* | TATAAGAGGTGACAAGAAAGT |
| Abi_fir_T1F1_pri# | GTAGCTCGCAAGGCTCATAACCTT |
| Abi_fir_T1R1_pri# | TCATTATGGATCGGATCAGTCCT |
| Abi_fir_T2F1_pri# | TTAGGTAGAGCTCCATCACCTT |
| Abi_fir_T2R1_pri# | TATCGTATTGGCCGATAAGCTCT |
| Ced_deo_T3F1_pri# | TCTGTCGAACAGGTATTGGAAGT |
| Ced_deo_T3R1_pri# | TATCCTTGATCGGTACTGCAGT |
| Cyc_rev_T10F1_pri# | TATTATTCACGAGGAGCCGGAT |
| Cyc_rev_T10R1_pri# | TCCAGATGTGATCCAAGCAGGTT |
| Cyc_rev_T10F2_pri# | CCGTGACTGCATAGGATTATAT |
| Cyc_rev_T11F1_pri# | TTGGCCTGGACTGGTCTAACAGAT |
| Cyc_rev_T11R1_pri# | CTGGCAATTGCTCAATGAACAT |
| Cyc_rev_T11R2_pri# | CTATGTCAACCGAGGTTCTAGAT |
| Pod_mac_T13F1_pri# | GCATTCCGCATCATTGTACAGAT |
| Pod_mac_T14F1_pri# | CAACGCCTAATGACTACCGCTATT |
| Pod_mac_T14R1_pri# | TGCGCTTGGATAATCCAATCCGTT |
| Pod_mac_T14R2_pri# | TTGCCTATCTAGAAGGTGCCTAACT |
| Pod_mac_T15F1_pri# | TTCGATCCTTCGTCGACGTAAGT |
| Pod_mac_T15F2_pri# | GTAGTTATGAATCCTGTGGACCAT |
| Pod_mac_T15R1_pri# | TGAGCAGACATACGTATCTGT |

| Primer name | Primer Sequences（5' to 3'） |
|---|---|
| Pod_mac_T15R2_pri# | CATCAGTTCCAGTATCATAAGTGCT |
| Tai_cry_T4F1_pri# | TGGAGAACTATAGCATTCCTCGCGT |
| Tai_cry_T4F2_pri# | GGCAATTGTTGCTTCTCTAGGAT |
| Tai_cry_T4R1_pri# | TCAGCCTTCGATGCAGAGTAT |
| Tax_cus_T5F1_pri# | TGGATAGCATGCTCCATAATGCAT |
| Tax_cus_T5F2_pri# | GCAGACATACGTATATGTCTAGCT |
| Tax_cus_T5R1_pri# | TCAATCCTTCGTCGACGTAAGTAGT |
| Tax_cus_T6F1_pri# | TCTATGTCCATCTGTGGTGTGACAT |
| Tax_cus_T6R1_pri# | CTGCTAGAAGTCTCTCTAGCAAGAT |
| Tax_cus_T12F1_pri# | TCAATAGATTCTGTTGGAGAG |
| Tax_cus_T12R1_pri# | TGAATGCTGGTTAATTGCATCT |
| Tax_cus_T12F2_pri# | TGCAAATGACAAGAGTATACTCTC |
| Tax_cus_T12R2_pri# | TATTCGATCTGAATGGACGAT |
| Wel_mir_T7F1_pri# | TAACAGCCAGTATCGTGGATCGT |
| Wel_mir_T7R1_pri# | GATAATGGCACCTGATACTT |
| Wel_mir_T7R2_pri# | GCCGAGTACTCTACCATTGAGTT |
| Wel_mir_T8F1_pri# | GCTTAGCAGACACCTTGGAACACAT |
| Wel_mir_T8R1_pri# | TAGCCGATAGAGCGGCGTATAAT |
| Wel_mir_T9F1_pri# | TTCCGAGATGAACTGGTAGATT |
| Wel_mir_T9R1_pri# | GCTGCTATTCAAGGTCGCATTGAT |
| Wel_mir_T9F2_pri# | TCCGCGATATAACGTCTCGTAC |
| Gne_1_R1_primer+ | TTGCCCTGCGAGATTTGTTGAT |
| Gne_2_F1_primer+ | ACAATACATTTCCTGGGTTCCT |
| Gne_2_R1_primer+ | ACCAAACCGAGTAACTGCCTAT |
| Gne_3_F1_primer+ | ATTTGTGCTGAATGCCTGTCCC |
| Gne_3_R1_primer+ | TCGACCGATCCTTCCCAGTTTACAT |
| Gne_4_F1_primer+ | GGCTAAACCCTATTTGTCCACT |
| Gne_4_R1_primer+ | TGGTTTGATAGTGGATTGCCTC |
| Gne_5_F1_primer+ | TAGAACATAATGATGCGACCCG |
| Gne_5_R1_primer+ | TACCCACGAACCTACTCTAACAAG |
| Gne_6_F1_primer+ | GTTCTACGGGAGATGCTTACTG |
| Gne_6_R1_primer+ | TTACGGTCAACAAAGGCGAAGC |
| Gne_7_F1_primer+ | CGGGCATTAGTTTAGAACCACG |
| Gne_7_R1_primer+ | CTGGAACTGCCATCCTAACTCT |
| Gne_8_F1_primer+ | AACCTCCACCGACATCTCCATT |
| Gne_8_R1_primer+ | CGCCCATGTATTGTTGATTCTG |
| Gne_9_F1_primer+ | TGGAATAACTCCCAATACAAGACTC |
| Gne_9_R1_primer+ | AACCCAAGAAATCCACAAACTG |
| Gne_9_F2_primer+ | CAACAAATCTCGCAGGGCAAAGT |
| Gne_9_R2_primer+ | GATTGAATCATATCGGTTACACT |
| Gne_9_F3_primer+ | TTCGACCTAACCGGACCTATT |
| Gne_9_R3_primer+ | GCAGCGTGTTTCCTCAAACT |
| Gne_10_F1_primer+ | ATTATGGGACAGGGTCGGATTC |

| Primer name | Primer Sequences（5’ to 3’） |
| --- | --- |
| Gne_10_R1_primer+ | AATCGGCAGCGTAAGTTCTCGT |
| Gne_11_F1_primer+ | TAAACGAGGTGCTCTACCGACT |
| Gne_11_R1_primer+ | CTACTATGCTCCAACCCGAAAT |
| Gne_11_F2_primer+ | CAGTGGTTAGAGTATCGCTTTC |
| Gne_11_R2_primer+ | CAATAGTGGCAAGATCGGTATAT |
| Gne_11_F3_primer+ | GACTGAGCTATAGCCCTGCGAT |
| Gne_11_R3_primer+ | TGACGCAGAAAAGGGTGCTGAT |
| Gne_12_F1_primer+ | CAAGTTTGAGTTTGCTCCAGTG |
| Gne_12_R1_primer+ | GATTTGCCGCTATTCCTTTCAC |
| Gne_12_F2_primer+ | TCCGGACCATGAGGTCAAGGGT |
| Gne_12_R2_primer+ | TGATTGGTTCTATCCAGAGCCT |
| Gne_13_F1_primer+ | CTTATACGGCAAGGGATGAATG |
| Gne_13_R1_primer+ | ACGATAATGGTGAACCCTCTAC |
| Gne_13_F2_primer+ | GTCACCCTTATACGGCAAGGGAT |
| Gne_13_R2_primer+ | TATTATGCAAGAGTGGTCCCGCT |
| Gne_14_F1_primer+ | AATGGTTATCGGTTCAAGGACG |
| Gne_14_R1_primer+ | CTCAATCCCAGGAAACAGTAAAG |
| Gne_14_R2_primer+ | TAGGTTCTGACGCAAGGTTAGAGT |
| Gne_15_R1_primer+ | GCTTTCAGTAGTTATCCGCTCC |
| Gne_16_F1_primer+ | TCATCTTGAGGTGGGCTTCTTA |
| Gne_16_R1_primer+ | CACCTGGACAGAAAGACCCTATG |
| Gne_16_F2_primer+ | CTAGAGTGGTATCTCACTGATGGCT |
| Gne_16_R2_primer+ | TACGGCGCTGAAGTAACCCACGTT |
| Gne_25_F1_primer+ | GTTCTACGGGAGATGCTTACTGT |
| Gne_25_R1_primer+ | TTACGGTCAACAAAGGCGAAGC |
| Gne_25_R2_primer+ | TGCTCTACTTCGATGGCTAGAAGACT |
| Gne_26_F1_primer+ | TACTCCAAGAAGCTGTAGATGCGCT |
| Gne_26_R1_primer+ | TGGGAACTTCTTGGTCTACTGAGGT |
| Gne_27_F1_primer+ | GGCATATGCCAGCTCTAACTGAGAT |
| Gne_27_R1_primer+ | GCTAAAGGATTTGCCGCTATTCCT |
| Gne_27_F2_primer+ | TCCAGTGGATACGGTGTAATTCAGT |
| Gne_27_R2_primer+ | CGAGAATATCGCGTTTTCCATG |
| Gne_28_F1_primer+ | CTGGACCAGTATAATCAGCCT |
| Gne_28_R1_primer+ | AAGGTGATAGGAACCAACCCT |
| Gne_28_F2_primer+ | GAAGGAGCGTTCTCCTGTATT |
| Gne_28_R2_primer+ | TGATGCCGTAGGTTCAAATCCT |
| Gne_28_F3_primer+ | TAGAATAGCCATGGAAACACGGAAT |
| Gne_28_R3_primer+ | GGCTAAAGATAACCTATGAGAGGT |
| Gne_28_F4_primer+ | CACGGAATCCAATATATAAGGT |
| Gne_28_R4_primer+ | TATAATTTGACGGAGAGGCGCGGGCT |
| Gne_29_F1_primer+ | TTCATCGGATTCCAGACGTGT |
| Gne_29_F2_primer+ | GCTCCAAACATATATGCCATGGT |
| Gne_29_R1_primer+ | ATCGATAGGTTCTGCCCAAT |

| Primer name | Primer Sequences（5' to 3'） |
|---|---|
| Gne_30_F1_primer+ | TTGGCAACCAGAGGCTAGTAAGTT |
| Gne_30_R1_primer+ | TACCACTCTAGAAGTGCCAGAACTCT |
| Gne_31_F1_primer+ | GGACCAGTGCTAGTAGTTCTT |
| Gne_31_F2_primer+ | TCCGGGTACTCACAGCTATTAGAAGT |
| Gne_31_R1_primer+ | TGCTATATAGCTGCGACCGAGTTGT |
| Gne_32_F1_primer+ | TAGGTATCCGAGGACTAATGTCAGAT |
| Gne_32_R1_primer+ | TCTCCAATAGACTGGCCTGCAAT |
| Gne_33_F1_primer+ | TGATCAGAACGAGTAGCGTCAGATGT |
| Gne_33_R1_primer+ | GCTGTATGCATTATATGCACGGCAAT |
| Gne_33_R2_primer+ | TGGTCCCATTTACGCTGATTAATCG |
| Gne_34_F1_primer+ | TCGATCTGTGACAATGTCCGACT |
| Gne_34_R1_primer+ | TAGGATCGATCAGTTTCCTCAGGACCT |
| Gne_35_F1_primer+ | TCTCTTCGACCGAGGAGATATCTCT |
| Gne_35_R1_primer+ | CTCGAAGATTGACGAAGGAACAATAGC |
| Gne_36_F1_primer+ | GTGGATCTGAAGCAGGTATTCCT |
| Gne_36_R1_primer+ | TTGAGCCTGAAGCTGCTATT |
| Gne_38_F1_primer+ | TAGTTGCTCTAGGTCAACCTCTTCGT |
| Gne_38_R1_primer+ | TACCGATGACTTTCGTGATGTAAGCT |
| Gne_39_F1_primer+ | TGCCAAGATATTGAATCTGTAGCAT |
| Gne_39_R1_primer+ | GTCAAACAAGTGGTCTGACTT |
| Gne_39_F2_primer+ | TAACACTTTAGCCAGTGCTTCCAGT |
| Gne_39_R2_primer+ | GTCCAAACTTCAGGATTATACGT |
| Gne_39_F3_primer+ | GATGATAAGTCATAACGATCAT |
| Gne_40_F1_primer+ | GCCAAGCCTATAAGGGTTGACT |
| Gne_40_R1_primer+ | CCATATCATTTGACTCTGGAT |
| Gne_40_F2_primer+ | TCAAGATTAAAGGAAAGATGGCCGAGC |
| Gne_41_F1_primer+ | CAAGGTGTATTCTTTCCACCTGCT |
| Gne_41_R1_primer+ | TTAAAGGTACCAAGCCTGCCTT |
| Gne_42_F1_primer+ | GTCACCCTTATACGGCAAGGGAT |
| Gne_42_R1_primer+ | TCTACATCTGACGCTACTCGTT |
| Gne_42_R2_primer+ | CTGGAAAGATAAAGAAACGGAAGAAGAT |
| T1F1_Ara_pri+ | GCGCTATTCGGATCTACACTTCTAGT |
| T1R1_Ara_pri+ | TGACATCATACAGGCGTGTGTT |
| T1R2_Ara_pri+ | TACGATGGACGTGGTACAATTCGT |
| T1F1_Cep_pri+ | GTAGACCGTGAGGACCAAGATGAAT |
| T1R1_Cep_pri+ | CGGTACAAGTGGCGTAGACAGTT |
| T2F1_Cep_pri+ | TATGGACCGAACTGTCTCACGACGTT |
| T2R1_Cep_pri+ | TGTCTGTGAAGATGCGGACTACCT |
| T3F1_Cep_pri+ | TACGCCATGCTACTGTGCCTTGTATGAT |
| T3R1_Cep_pri+ | GCTGCACAATGCCGATCTTCAT |
| T1F1_Cun_pri+ | TCCGGCTTAACACCGATAGTCTGTT |
| T1R1_Cun_pri+ | CGATGGATACTAAGTGCTGTGCGTAT |
| T2F1_Cun_pri+ | CCATCTTGGAGCAGTGCTATGCTTAGT |

| Primer name | Primer Sequences（5’ to 3’） |
|---|---|
| T2R1_Cun_pri+ | TGCAATCACAGGTTCCATCGT |
| T2F2_Cun_pri+ | GAATCGGCTATGCAAGGTAGAGT |
| T1F1_Cyc_pri+ | TATGGACCGAACTGTCTCACGACGTT |
| T1R1_Cyc_pri+ | TGAAGATGCGGACTACCTGCACCT |
| T2F1_Cyc_pri+ | TCCAGTACGGCTACCTTGTTACGACT |
| T2R1_Cyc_pri+ | GAGGAATAAGCATCGGCTAACTCTGT |
| T3F1_Cyc_pri+ | TACTTCGTATTGCCGAGATCCAAGGAT |
| T3R1_Cyc_pri+ | TCGCCATCCTATCCAATGGATGAT |
| T4F1_Cyc_pri+ | TCCGAGGAATAAGCATCGGCTAACT |
| T4R1_Cyc_pri+ | TCCAGTACGGCTACCTTGTTACGACT |
| T5F1_Cyc_pri+ | TGAAGATGCGGACTACCTGCACCT |
| T5R1_Cyc_pri+ | TATGGACCGAACTGTCTCACGACGTT |
| T1F1_Eph_pri+ | GAACTCTAACCTTGCGTCAGAACCT |
| T1R1_Eph_pri+ | CGTGACCTTATCGGTTACTCGAT |
| T2F1_Eph_pri+ | TTCAGTAGTTATCCGCTCCGCACTT |
| T2R2_Eph_pri+ | TGGAGCCATCAGTGAGATACCACTCT |
| T1F1_Gin_pri+ | TGTCTGTGAAGATGCGGACTACCT |
| T1R1_Gin_pri+ | TCCACTTGACACCTATCGTGATGAT |
| T2F1_Gin_pri+ | TCTCGCCGTGACCTTATCTTGGATT |
| T2R2_Gin_pri+ | TATCGGTTCAAGGACGCAAGGTGACCT |
| T3F1_Gin_pri+ | GGGGAATGTGTGAAGAAGGGAC |
| T3R1_Gin_pri+ | CTACTCGTTGACCCCCTCGTTT |
| T1F1_Pin_arm_pri+ | TCAAGTCATGCGTTGGTACGATACT |
| T1R1_Pin_arm_pri+ | CGTCAGTAGCATATTTCTATGCAGGT |
| T2F1_Pin_arm_pri+ | TGATCTGTCCAGTGCTTATGTATCC |
| T2R1_Pin_arm_pri+ | TTAGCATCTGTCATTCTGGCACAGAT |
| T3F1_Pin_arm_pri+ | TACAATTAATCCGGCATGAGCCACGT |
| T3R1_Pin_arm_pri+ | TCATGTTATTTGTGCCAGTGACCGGT |
| T4F1_Pin_arm_pri+ | TGGTTCTAATGATGATCCTGCACGT |
| T4R1_Pin_arm_pri+ | GCATGGCCTAATCAATAGTTCAAGT |
| T5F1_Pin_arm_pri+ | GGAAGTAATTCGTTGAGATCATGCACT |
| T5R1_Pin_arm_pri+ | TGCGGGAGAGGCTGATCTTAATAATT |
| T5R2_Pin_arm_pri+ | TCTGTCCATTAGATCTCCAGATAGAGT |
| T6F1_Pin_arm_pri+ | TCCTATCTTCAACAAGTGGTCCAGCAT |
| T6R1_Pin_arm_pri+ | CGAGAGTTTCAATCAGTTCCTGCCAAT |
| T7F1_Pin_arm_pri+ | TAGATTGTCCACGCTAACGATCCATCCT |
| T7R1_Pin_arm_pri+ | TCACGTGGCTCATGCCGGATTAATT |
| T8F1_Pin_arm_pri+ | GGACTGATCCGATCCATAATGAT |
| T8R1_Pin_arm_pri+ | GGATCAGATGGTATAGTCTATTCAATC |
| T8R2_Pin_arm_pri+ | TCAATGCGGTGAATTATGTCTCTCC |
| T9F1_Pin_arm_pri+ | TCATGATGATGCCATGTGAATTGCT |
| T9R1_Pin_arm_pri+ | TACTTCGCCGTGACATGAGTGT |
| T10F1_Pin_arm_pri+ | TGTGCGAACCAATGGGAACGATCAT |

| Primer name | Primer Sequences（5’ to 3’） |
| --- | --- |
| T10R1_Pin_arm_pri+ | TCGTGGTCGTTCATATGGACAAGCACT |
| T11F1_Pin_arm_pri+ | TGAGGCTATTCCTGTAATAGGATCTCCT |
| T11R1_Pin_arm_pri+ | CTGGCGATGATATATTACCGTCGTTACT |
| T12F1_Pin_arm_pri+ | CGACGAAGGATTGAAGCATCACTGT |
| T12R1_Pin_arm_pri+ | CTGAGAGAGGCTTGTACAGTT |
| T1F1_Pod_pri+ | TATCACCGATGGTCTGTTCAGAGTT |
| T1R1_Pod_pri+ | GGATGCAAGCGTTATCCGGAATTAT |
| T1F1_Pic_smi_pri+ | TCTATGGCTACACAGACCATTGAT |
| T1R1_Pic_smi_pri+ | TATCCAACGAGGTAGGACCATCTT |
| T2F1_Pic_smi_pri+ | TGCAACAGGTACGCCAATCACTAGT |
| T2R1_Pic_smi_pri+ | TACAGCCTTGGCAGGAACGACGTT |
| T3F1_Pic_smi_pri+ | GTCAAGGGGAATGTGTAAAGAAG |
| T3R1_Pic_smi_pri+ | TCCATTTCTCCTATTCGTTGCCC |
| T1F1_Tai_pri+ | TGATACAGATTGTGATCCAGATCTCGT |
| T1R1_Tai_pri+ | CAGGAAGTTATAAGTACCTGTGCATGAACT |
| T1R2_Tai_pri+ | TCCTAGGAACATTACAGGTATAGGTACCACT |
| T2F1_Tai_pri+ | TATGGACCGAACTGTCTCACGACGTT |
| T1F1_Tax_pri+ | TCTAACCTTGCGTCAGAACCTACTGT |
| T1R1_Tax_pri+ | TTATGGAGAGCACTCATCTTGAGGT |
| T2F1_Tax_pri+ | GGAAACATTATCTATCTCGGCACT |
| T2R1_Tax_pri+ | ACCCCTTGATTTAATGCCAGAT |
| T1F1_Wel_pri+ | TGCCAGAACTCTAACCTTGCGT |
| T1R1_Wel_pri+ | TATGGACCGAACTGTCTCACGACGTT |
| T2F1_Wel_pri+ | TATGGACCGAACTGTCTCACGACGTT |
| T2R1_Wel_pri+ | CATGTCTGTGAAGATGCGGACTACCT |
| T3F1_Wel_pri+ | TGCACACTTAGATGCTACTACCGTACT |
| T3R1_Wel_pri+ | TTCCTGAGCCTCTTGAGGATCAAT |
| T1F1_Zam_pri+ | TAGAGCCAGTAGACGAATACGACAT |
| T2F1_Zam_pri+ | TAGACATGTCTGTGAAGATGCGGACT |
| T2R1_Zam_pri+ | TGCACTTCCACTTGACACCTATCGTGAT |
| T3F1_Zam_pri+ | TATGGACCGAACTGTCTCACGACGT |
| T3R1_Zam_pri+ | TCTCCGCGAAGTCGTAAGACCATGTAT |
| T4F1_Zam_pri+ | TGCCAGAGTACGATTAACCTCTGGAT |
| T4R1_Zam_pri+ | GATCCACCTACACGTCTTGCTT |
| T4F2_Zam_pri+ | TCGTCAACAAGGGCGTTCTAGTGCGT |
| T4R2_Zam_pri+ | TAATGACCAACGAATGGCAAGTGCTT |

- used for gap filling

* used directly for sequencing

# used for verification after filling gaps

+ used for RT-PCR
